# Ionization of HCCI Neutral and Cations by Strong Laser Fields Simulated With Time Dependent Configuration Interaction

**DOI:** 10.3389/fchem.2022.866137

**Published:** 2022-04-25

**Authors:** H. Bernhard Schlegel, Paul Hoerner, Wen Li

**Affiliations:** Department of Chemistry, Wayne State University, Detroit, MI, United States

**Keywords:** HCCI+, iodoacetylene cation, strong field ionization, coherent superposition, time-dependent configuration interaction, pump-probe

## Abstract

Strong field ionization of neutral iodoacetylene (HCCI) can produce a coherent superposition of the X and A cations. This superposition results in charge migration between the CC *π* orbital and the iodine *π*-type lone pair which can be monitored by strong field ionization with short, intense probe pulses. Strong field ionization of the X and A states of HCCI cation was simulated with time-dependent configuration interaction using singly ionized configurations and singly excited, singly ionized configurations (TD-CISD-IP) and an absorbing boundary. Studies with static fields were used to obtain the 3-dimensional angular dependence of instantaneous ionization rates by strong fields and the orbitals involved in producing the cations and dications. The frequency of charge oscillation is determined by the energy separation of the X and A states; this separation can change depending on the direction and strength of the field. Furthermore, fields along the molecular axis can cause extensive mixing between the field-free X and A configurations. For coherent superpositions of the X and A states, the charge oscillations are characterized by two frequencies–the driving frequency of the laser field of the probe pulse and the intrinsic frequency due to the energy separation between the X and A states. For linear and circularly polarized pulses, the ionization rates show marked differences that depend on the polarization direction of the pulse, the carrier envelope phase and initial phase of the superposition. Varying the initial phase of the superposition at the beginning of the probe pulse is analogous to changing the delay between the pump and probe pulses. The charge oscillation in the coherent superposition of the X and A states results in maxima and minima in the ionization yield as a function of the superposition phase.

## Graphical Abstract

Time-dependent configuration interaction simulations of the electron dynamics in coherent superpositions of the X and A cation states of HCCI show marked differences in the strong field ionization rates by intense, 2 cycle probe pulses that depend on the initial phase of the superposition and on the carrier envelope phase.
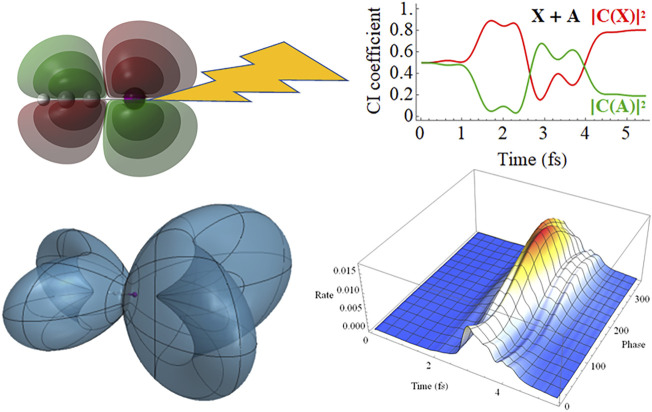

## Introduction

Producing and probing ultrafast electronic wave packets are important topics of attosecond spectroscopy and have attracted much attention in the past decade. A few atomic and molecular systems have been shown to exhibit coherent electronic motions in the few to tens of femtoseconds range. (Smirnova et al., 2009; Goulielmakis et al., 2010; Calegari et al., 2014; Kraus et al., 2015). Various experimental techniques have been developed to achieve excellent temporal resolution and these include attosecond transient absorption, attosecond pump-IR probe, high harmonic spectroscopy, and attosecond streaking. (Kienberger et al., 2004; Eckle et al., 2008; Smirnova et al., 2009; Goulielmakis et al., 2010; Calegari et al., 2014; Winney et al., 2017). Strong field ionization (SFI) plays an important role in most of these developed techniques owing to its sub-cycle timing arising from the high nonlinearity of the process. SFI has been used either as a pump pulse to excite the electronic superposition or as a probe to detect the wavepacket motion. A simple extrapolation would suggest it might be possible to employ SFI in both steps to produce and probe electronic dynamics. In this article, computational simulations were carried out to explore SFI of a molecule undergoing coherent electronic dynamics. Iodoacetylene, HCCI, is selected as the model system. The superposition between the X and A states of HCCI cation has been studied previously using high harmonic spectroscopy and advanced modeling. (Kraus et al., 2015; Ding et al., 2017; Jenkins et al., 2018; Jia et al., 2019a; Jia et al., 2019b; Jia and Yang, 2022). However, due to the limited probing range of HHS, the wave packet motion beyond the first three femtoseconds has not been studied. Recent theoretical investigations suggested the coherence will rapidly dephase after 5 fs and even rephase at a later time. (Jia et al., 2019a; Jia et al., 2019b; Jia and Yang, 2022). Therefore, a new probing method with attosecond time resolution and a long probing range is needed. Here we show theoretically that SFI and second ionization implemented with few-cycle laser pulses can indeed follow the electronic wave packet motion and thus offer a long range attosecond probing method. Furthermore, our investigation reveals detailed and complex electronic dynamics associated with a SFI probe, which involves laser driven dynamics as well as intrinsic electronic dynamics.

Recent reviews discuss theoretical and computational methods for describing electron dynamics and ionization in strong laser fields. (Posthumus, 2004; Ishikawa and Sato, 2015; Nisoli et al., 2017; Goings et al., 2018; Li et al., 2020; Palacios and Martin, 2020). One and two electron systems can be treated accurately by solving the time dependent Schrodinger equation. For multi-electron systems, the single active electron (SAE) approximation and the strong field approximation (SFA) are often used. Orientation dependent ionization rates can be modelled with molecular Ammosov-Delone-Krainov (Tong et al., 2002) (MO-ADK) and weak-field asymptotic theory (Tolstikhin et al., 2011) (WFAT). More detailed descriptions of ionization by intense laser fields require numerical simulations of the electron dynamics. Methods for time dependent electronic structure methods have been reviewed recently. (Nisoli et al., 2017; Goings et al., 2018; Li et al., 2020; Palacios and Martin, 2020). Two approaches that have been used successfully to simulate strong field ionization for multi-electron polyatomic systems are real-time integration of time-dependent density functional theory (Chu and Chu, 2001; Chu, 2010; Hellgren et al., 2013; Lopata and Govind, 2013; Provorse and Isborn, 2016; Bruner et al., 2017; Sandor et al., 2018) (rt-TDDFT) and time-dependent configuration interaction (TD-CI). (Krause et al., 2005; Rohringer et al., 2006; Krause et al., 2007; Klinkusch et al., 2009; Greenman et al., 2010; Tremblay et al., 2011; Krause et al., 2014; Krause and Schlegel, 2015a). In these approaches, ionization is treated by removing the outgoing electron density using a complex absorbing potential. (Kosloff and Kosloff, 1986; Santra and Cederbaum, 2002; Muga et al., 2004; Krause et al., 2014; Krause and Schlegel, 2015a; Sommerfeld and Ehara, 2015).

In previous work, we have used time-dependent configuration interaction with a complex absorbing potential to study strong field ionization. (Krause et al., 2014; Krause and Schlegel, 2015a; Krause and Schlegel, 2015b; Hoerner and Schlegel, 2017; Hoerner and Schlegel, 2018; Winney et al., 2018; Lee et al., 2020). In particular, we have used TD-CIS to examine the angular dependence of strong field ionization of haloacetylenes, HCCX (X = F, Cl, Br, and I). (Hoerner and Schlegel, 2018). The present study examines some aspects of coherent electron dynamics in HCCI cations that can be generated by intense laser pump pulses and probed by strong field ionization using intense,ultra-short pulses. To provide some background of the electronic behavior of HCCI^+^ in intense fields, the angular dependence of strong field ionization of the X and A states are studied with a static field and fixed nuclei. Next, the coherent superpositions of the X and A states of the cation are examined for the field free case and for strong field ionization by a static field. The purpose of the static field studies is to help understand the electronic response HCCI cations in strong fields. The time-dependent electron dynamics for strong field ionization by very short linearly and circularly polarized probe pulses are simulated for the X and A states of HCCI cation and their coherent superpositions. Nuclear dynamics leads to dephasing which modulates the electron dynamics. (Ding et al., 2017; Jenkins et al., 2018; Jia et al., 2019a; Jia et al., 2019b; Jia and Yang, 2022). This is addressed in a separate paper by Jia and Yang in this collection of articles on electronic and nuclear dynamics of molecules in intense laser fields. (Jia and Yang, 2022). The focus of the present paper is on the use of strong field ionization to probe the electron dynamics of HCCI^+^. To minimize the effects of decoherence caused by nuclear motion during the pulse, the probe pulses are limited to two cycles (2.66 fs FWHM).

## Computational Methods

The electronic wavefunction is propagated with the time-dependent Schrödinger equation (atomic units are used throughout the paper).

$$i\hbar \frac{\partial }{\partial t}\text{Ψ}\left(t\right)=\hat{\text{H}}\left(t\right)\text{Ψ}\left(t\right)=\left[{\hat{\text{H}}}_{el}-\hat{\vec{\mu }}\cdot \vec{E}\left(t\right)-i\;{\hat{\text{V}}}^{absorb}\right]\text{Ψ}\left(t\right)$$ (1)

${\hat{\text{H}}}_{el}$ is the field-free non-relativistic electronic Hamiltonian. The interaction with the intense electric field is treated in the semiclassical dipole approximation, where $\hat{\vec{\mu }}$ is the dipole operator and $\vec{E}$ is the electric field. Ionization is modeled with a complex absorbing potential (CAP), -*i* **V** ^*absorb*^, as described in our earlier papers. (Krause et al., 2014; Krause and Schlegel, 2015a; Krause and Schlegel, 2015b; Hoerner and Schlegel, 2017; Hoerner and Schlegel, 2018; Winney et al., 2018; Lee et al., 2020) The total absorbing potential for the molecule is equal to the minimum of the values of spherical absorbing potentials centered on each atom. Each spherical potential begins at 3.5 times the van der Waals radius of each element (*R*_H_ = 9.544 bohr, *R*_C_ = 12.735 bohr, *R*_I_ = 14.882 bohr), rises as sin((π/2)(*R–R*_*0*_)/(*R*_*1*_ *-R*_*0*_))^2^ to 10 hartree at approximately *R*_*1*_ = *R*_*0*_ + 28 bohr and is equal to 10 hartree for *R* > *R*_*1*_. The decrease in the norm^2^ of the wavefunction is taken as the total ionization yield. The instantaneous ionization rate is calculated as the rate of decrease in the norm^2^ and can be related to the matrix elements of the absorbing potential.

$$\text{rate}\left(t\right)=-\partial \langle {\text{Ψ}}_{neutral}\left(t\right){|\Psi }_{neutral}\left(t\right)\rangle /\partial \;t=\frac{2}{\hbar }\langle {\text{Ψ}}_{neutral}\left(t\right){\mathrm{|V}}^{absorb}{\text{|Ψ}}_{neutral}\left(t\right)\rangle$$ (2)

The matrix elements of **V** ^*absorb*^ can be written in terms of the molecular orbitals to give the contribution of individual molecular orbitals to the total ionization rate. (Lee et al., 2020).

For simulations of the ionization of neutral HCCI with TD-CIS, the wavefunction includes the Hartree-Fock reference determinant and all distinct *α→α* and *β→β* single excitations from the active orbitals,

$${\text{Ψ}}_{neutral}\left(t\right)=\sum_{I=0}C_{I}\left(t\right)|{\text{Ψ}}_{I}\rangle =c_{0}{\text{Ψ}}_{0}+\sum_{ia}c_{i}^{a}{\text{Ψ}}_{i}^{a}+\sum_{\bar{i}\bar{a}}c_{\bar{i}}^{\bar{a}}{\text{Ψ}}_{\bar{i}}^{\bar{a}}$$ (3)

where *β* orbitals are indicated by an overbar. Simulations of the ionization of HCCI cations were carried out with TD-CISD-IP. (Lee et al., 2020) The CISD-IP wavefunction (Golubeva et al., 2009) includes singly ionized determinants, ${\text{Ψ}}_{x}$ , constructed by removing an electron from each of the active orbitals of the neutral molecule and all *α→α* and *β→β* single excitations from these determinants,

$$\begin{array}{l} {\text{Ψ}}_{cation}\left(t\right)=\sum_{I=0}C_{I}\left(t\right)|{\text{Ψ}}_{I}\rangle =\sum_{x}c_{x}{\text{Ψ}}_{x}+\sum_{\bar{x}}c_{\bar{x}}{\text{Ψ}}_{\bar{x}}+\\ \;\;\;\;\;\;\;\;\;\;\;\;\;\;\;\;\;\;\;\;\sum_{iax}c_{ix}^{a}{\text{Ψ}}_{ix}^{a}+\sum_{ia\bar{x}}c_{i\bar{x}}^{a}{\text{Ψ}}_{i\bar{x}}^{a}+\sum_{\bar{i}\bar{a}x}c_{\bar{i}x}^{\bar{a}}{\text{Ψ}}_{\bar{i}x}^{\bar{a}}+\sum_{\bar{i}\bar{a}\bar{x}}c_{\bar{i}\bar{x}}^{\bar{a}}{\text{Ψ}}_{\bar{i}\bar{x}}^{\bar{a}} \end{array}$$ (4)

The hole density for the cation is the difference of the one electron densities of the neutral and the cation. Movies of the hole density are useful for examining the electron dynamics in a laser field. Aspects of the electron dynamics of the cations can also be followed by looking at occupied-occupied elements of the density matrix (for the field strengths considered here, the other elements of the cation density matrix are much smaller). For a CISD-IP wavefunction, the occupied-occupied block of the one electron density matrix in terms of molecular spin orbitals is given by

$$\rho_{i,j}^{occ}=\delta_{i,j}-c_{j}^{*}c_{i}-\sum_{ka}c_{jk}^{a\;*}c_{ik}^{a}$$ (5)

As in our previous studies, (Krause et al., 2014; Krause and Schlegel, 2015a; Krause and Schlegel, 2015b; Hoerner and Schlegel, 2017; Hoerner and Schlegel, 2018; Winney et al., 2018; Lee et al., 2020), the exponential of the Hamiltonian is used to propagate the time-dependent wavefunction. For a linearly polarized pulse, a Trotter factorization is employed to compute the exponential.

$$\begin{array}{l} \text{Ψ}\left(t+\text{Δ}t\right)=\exp(-i\;\hat{H}\text{Δ}t)\text{Ψ}\left(t\right)\\ C\left(t+\text{Δ}t\right)=\exp(-i\;H_{el}\text{Δ}t/2)\exp(-V^{absorb}\text{Δ}t/2)\\ \;\;\;\;\;\;\;\;\;\;\;\;\;\;\;\;\;\;\;\;\;\;\;\;\;\;\;\;\times W^{T}\exp\left(i\;E\left(t+\text{Δ}t/2\right)\;d\;\Delta t\right)\;W\\ \;\;\;\;\;\;\;\;\;\;\;\;\;\;\;\;\;\;\;\;\;\;\;\;\;\;\;\;\times \exp(-V^{absorb}\text{Δ}t/2)\exp(-i\;H_{el}\text{Δ}t/2)C\left(t\right) \end{array}$$ (6)

**WDW** ^*T*^ = **d** are the eigenvalues and eigenvectors of the transition dipole matrix **D** in the field direction. The matrices $\exp\left(-i\;H_{el}\text{Δ}t/2\right)$ , $\exp\left(-V^{absorb}\text{Δ}t/2\right)$ , **W** and **d** need to be calculated only once at the beginning of the propagation because they are time independent. Likewise, the product **U** = $\exp\left(-V^{absorb}\text{Δ}t/2\right)$ **W** ^***T***^ is formed once at the beginning of the propagation. The only time dependent factor is exp(i E(t+Δt/2) d Δt) ; this exponential can be calculated easily because **d** is a diagonal matrix. A propagation step for a linearly polarized pulse with fixed nuclear positions involves two full matrix-vector multiplies (**U** and **U** ^*T*^) and three diagonal matrix-vector multiplies ( $\exp\left(-i\;H_{el}\text{Δ}t/2\right)$ and exp(i E(t+Δt/2) d Δt) ).

The corresponding Trotter factorization for a circularly polarized pulse involves two oscillating fields

$$\begin{array}{l} C\left(t+\text{Δ}t\right)=\exp(-i\;H_{el}\text{Δ}t/2)\exp(-V^{absorb}\text{Δ}t/2)\\ \;\;\;\;\;\;\;\;\;\;\;\;\;\;\;\;\;\;\;\;\;\;\;\;\;\;\;\;\times W_{2}^{T}\exp\left(i\;E_{2}\left(t+\text{Δ}t/2\right)\;d_{2}\;\text{Δ}t/2\right)\;W_{2}\\ \;\;\;\;\;\;\;\;\;\;\;\;\;\;\;\;\;\;\;\;\;\;\;\;\;\;\;\;\times W_{1}^{T}\exp\left(i\;E_{1}\left(t+\text{Δ}t/2\right)\;d_{1}\;\text{Δ}t\right)\;W_{1}\\ \;\;\;\;\;\;\;\;\;\;\;\;\;\;\;\;\;\;\;\;\;\;\;\;\;\;\;\;\times W_{2}^{T}\exp\left(i\;E_{2}\left(t+\text{Δ}t/2\right)\;d_{2}\;\text{Δ}t/2\right)\;W_{2}\\ \;\;\;\;\;\;\;\;\;\;\;\;\;\;\;\;\;\;\;\;\;\;\;\;\;\;\;\;\times \exp(-V^{absorb}\text{Δ}t/2)\exp(-i\;H_{el}\text{Δ}t/2)C\left(t\right) \end{array}$$ (7)

where **W**_1_ **D**_1_ **W**_1_^*T*^ = **d**_1_ and **W**_2_ **D**_2_ **W**_2_^*T*^ = **d**_2_ are the eigenvalues and eigenvectors of the transition dipole matrix **D**_1_ and **D**_2_ in the two orthogonal field directions. A propagation step for a circularly polarized pulse with fixed nuclei involves four full matrix-vector multiplies and five diagonal matrix-vector multiplies.

The present methodology has been tested in an earlier paper (Krause et al., 2014) and satisfactory agreement was obtained in comparisons with the results for ionization of hydrogen atom and H_2_^+^ calculated with grid-based methods. (Hehenberger et al., 1974; Hermann and Fleck, 1988; Bandrauk et al., 2012). While grid-based methods are limited to one and two electron systems, the TDCI approach can be applied to many-electron, polyatomic molecules. Unlike the strong field approximation (SFA) and single active electron (SAE) approximation, the TDCI calculations include exchange interactions of the ionizing electron, and the dynamics of the remaining valence electrons. Because the propagation uses the exponential of the Hamiltonian, a fairly large time step of Δ*t* = 0.05 au (1.2 as) can be used. In similar simulations, reducing the time step by a factor of 2 changed the ionization yield by less than 0.01%. (Hoerner and Schlegel, 2017). Once the initial eigenvectors and matrix exponentials are calculated, the cost of the propagation steps is O(N^2^) compared to O(N^3^) for real-time integration of TD-DFT. (Li et al., 2020).

Directional information for ionization was obtained by examining the ionization rate in a static field. Abruptly turning on a strong field can cause extensive non-adiabatic excitation. A practical approach to avoid non-adiabatic excitations involves ramping up the electric field slowly and calculating the instantaneous ionization rate when the field has reached a constant value. (Hermann and Fleck, 1988). The instantaneous ionization rate was calculated after 800 au (19.35 fs) using

$$E\left(t\right)=E_{\max}\left(1-{\left(1-\frac{t}{t_{ramp}}\right)}^{4}\right)\text{for }\;0\leq t\leq t_{ramp},E\left(t\right)=E_{\max}\text{for}\;t\geq t_{ramp}$$ (8)

with *t*_*ramp*_ = 533 au = 12.90 fs.

Simulations of strong field ionization of HCCI cations with linearly polarized light used a 2 cycle 800 nm (ω = 0.057 au) pulse in the xz plane with a sin^2^ envelope,

$$E\left(t\right)=E_{\max}\sin{\left(\omega \;t/4\right)}^{2}\cos\left(\omega \;t\right)\text{for}\;0\leq \omega \;t\leq 4\pi ,E\left(t\right)=0\;\text{for}\;\omega \;t\geq \;4\pi$$ (9)

for HCCI aligned with the *z* axis. Simulations with circularly polarized light used a 2 cycle 800 nm pulse in the xz plane with a sin^2^ envelope (FWHM = 2.66 fs).

$$\begin{array}{l} E_{x}\left(t\right)=E_{\max}\sin{\left(\omega \;t/4\right)}^{2}[-\cos\left(\omega \;t\right)\cos\left(\gamma \right)-\sin\left(\omega \;t\right)\sin\left(\gamma \right)]\\ E_{z}\left(t\right)=E_{\max}\sin{\left(\omega \;t/4\right)}^{2}\left[\cos\left(\omega \;t\right)\sin\left(\gamma \right)-\sin\left(\omega \;t\right)\cos\left(\gamma \right)\right]\\ \;\;\;\;\;\text{for }\;0\leq \omega \;t\leq 4\pi ,\;\;E_{z}\left(t\right)=E_{x}\left(t\right)=0\;\text{for}\;\omega \;t\geq 4\pi \;\;\;\;\; \end{array}$$ (10)

**FIGURE 1:**
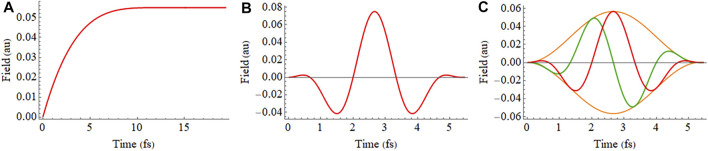
**(A)** Shape of the electric field used to obtain the instantaneous ionization in a static field (Eq. 8), **(B)** 2 cycle linearly polarized 800 nm pulse with a sin^2^ envelope (Eq. 9), and **(C)** 2 cycle circularly polarized 800 nm pulse with a sin^2^ envelope (Eq. 10) showing x and z components in green and red, respectively.

*E*_*max*_ is the maximum value for the electric field and *γ* determines the direction of the field at the maximum of the pulse. The electric fields for Eqs 8–10 are shown in Figure 1.

A locally modified version of the Gaussian software package (Frisch et al., 2019) was used to calculate the CAP integrals needed for the TDCI simulation. The TDCI simulations were carried out with an external Fortran95 code. Mathematica (Mathematica 12, 2019) was used to analyze the simulations and plot the results. Movies were prepared with VMD. (Humphrey et al., 1996). The CC, CH, and CI bond lengths used for HCCI were 1.0542, 1.1819, and 1.9982 Å, respectively. HCCI was placed on the *z* axis with the iodine in the -z direction. All of the simulations were carried out with fixed nuclei. The aug-cc-pVTZ basis set (Dunning, 1989; Woon and Dunning, 1993; Peterson et al., 2006) was used for H and C; the aug-cc-pVTZ-PP basis set with pseudopotential was used for iodine. (Peterson et al., 2003). These basis sets were augmented with a set of additional diffuse functions placed on each atom to describe the electron dynamics during the ionization process and to ensure adequate interaction with the CAP. (Krause et al., 2014; Hoerner and Schlegel, 2017). This “absorbing basis” consisted of four *s* functions with exponents of 0.0256, 0.0128, 0.0064, and 0.0032; four *p* functions with exponents of 0.0256, 0.0128, 0.0064, and 0.0032; five *d* functions with exponents of 0.0512, 0.0256, 0.0128, 0.0064, and 0.0032; and two *f* function with an exponent of 0.0256 and 0.0128. The time-dependent wavefunction for HCCI included all excitations from the highest σ orbital and two highest π and π* orbitals to all virtual orbitals with orbital energies less than 3 hartree, for a total of 2,621 configurations for HCCI neutral and 18,530 configurations for HCCI cations. For studies with a static field, *E*_max_ = 0.033 au was optimal for neutral HCCI and 0.055 au for the cation, while *E*_max_ = 0.075 au and 0.080/ $\sqrt{2}$ = 0.0566 au were used for ionization of the cation with linear and circularly polarized pulses, respectively. Smaller values of *E*_max_ yielded too little ionization of the neutral and the X state of the cation; larger values lead to artifacts in the angular dependence of the ionizations of the cations.

## Results and Discussion

**FIGURE 2:**
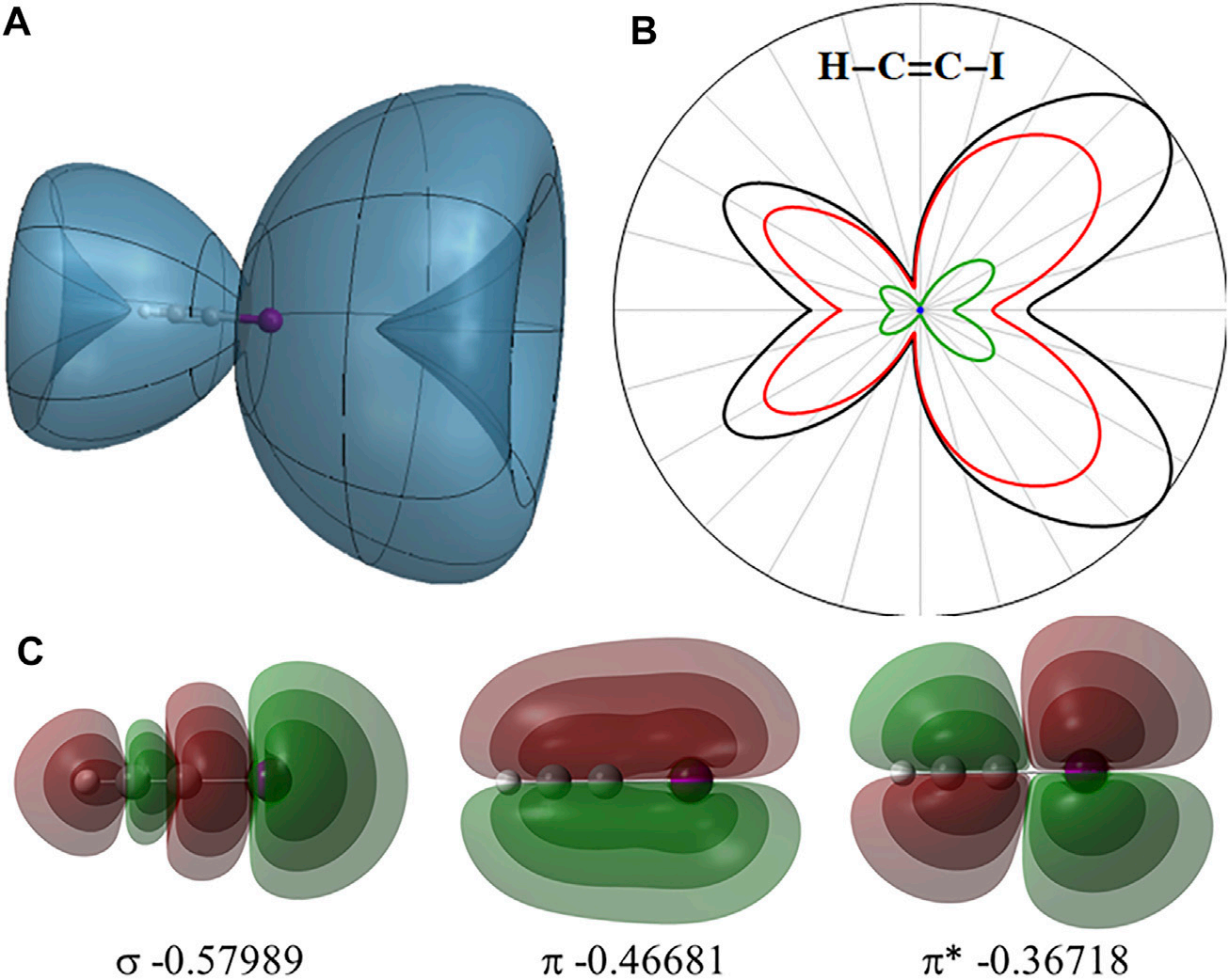
**(A)** Angular dependence of the instantaneous ionization rate for neutral HCCI in static field of 0.033 au. **(B)** 2-Dimensional slice showing the total ionization rate (black) and contributions from the σ, π , and $\pi^{*}$ orbitals (blue, green and red, respectively). **(C)** Highest occupied *σ*, π and $\pi^{*}$ orbitals of HCCI and their orbital energies (in hartree).

The angular dependence of the instantaneous ionization rate of neutral HCCI in a static field obtained with TD-CIS simulations is shown in Figure 2 along with the highest occupied *σ*, π and $\pi^{*}$ orbitals of HCCI. The π and $\pi^{*}$ orbitals are the in-phase and out-of-phase combinations of the CC *π* orbitals and the iodine *π*-type lone pairs. When the field has reached a constant value, the shape of the instantaneous ionization rate and the total ionization yield both resemble the nodal structure of the highest occupied $\pi^{*}$ orbital. Similar shapes were found with WFAT calculations. (Tolstikhin et al., 2011). Partitioning the instantaneous ionization rate into orbital contributions (Lee et al., 2020) shows that ionization is predominantly from the $\pi^{*}$ orbital leading to the ground state of the cation, the X state (see Figure 2B). The π orbital is 0.1 hartree lower in energy and contributes to a lesser extent. Removal of an electron from this orbital leads to the lowest excited state of the cation (the A state). The iodine *σ*-type lone pair orbital is 0.2 hartree lower in energy than the $\pi^{*}$ orbital and does not contribute significantly to ionization at this field strength. Ionization of HCCI by a strong field can result in a coherent superposition of cation states, mainly the X and A states. The ratio of the calculated ionization rates of the X and A states is 3.9 when averaged over the angles, in good agreement with the experimental ratio of 4.3 for the populations of the superposition of the X and A states found in the analysis of the HHG spectra. (Kraus et al., 2015).

The experimental energies for the vertical ionization from the neutral to the X and A states of the cation are 9.71 and 11.94 eV, respectively. (Allan et al., 1977). Ionization energies calculated by Koopmans theorem are just the negative of the orbital energies (9.99 and 12.70 eV, resp.); these values are in reasonably good agreement with experiment because of a cancellation of errors caused by the neglect of orbital relaxation and electron correlation effects. Electron propagator theory (Ortiz, 1996) (EPT) treats both relaxation and correlation, resulting in improved ionization potentials (9.89 and 12.22 eV, resp. with the aug-cc-pVTZ-PP basis set). EOMIP/CCSD calculations give even better agreement with experiment. (Kraus et al., 2015). However, EPT and EOMIP/CCSD cannot be used in TDCI simulations of the cations since thousands of excited states are needed to model the electron dynamics of strong field ionization. Spin unrestricted CIS could be employed, but this results in different orbitals for the X and A states. As an alternative, the TD-CISD-IP approach can be used to treat the dynamics of the ground and excited states of the cation on an equal footing. The CISD-IP ionization energies (8.55 and 10.76 eV with aug-cc-pVTZ-PP plus the absorbing basis) are about an eV too low because they include some orbital relaxation but little correlation. However, the difference in the energies of the X and A states is the most important factor for the dynamics of a superposition of these states. The difference in the CISD-IP ionization energies, 2.21 eV, is in very good agreement with the experimental difference, 2.23 eV.

**FIGURE 3:**
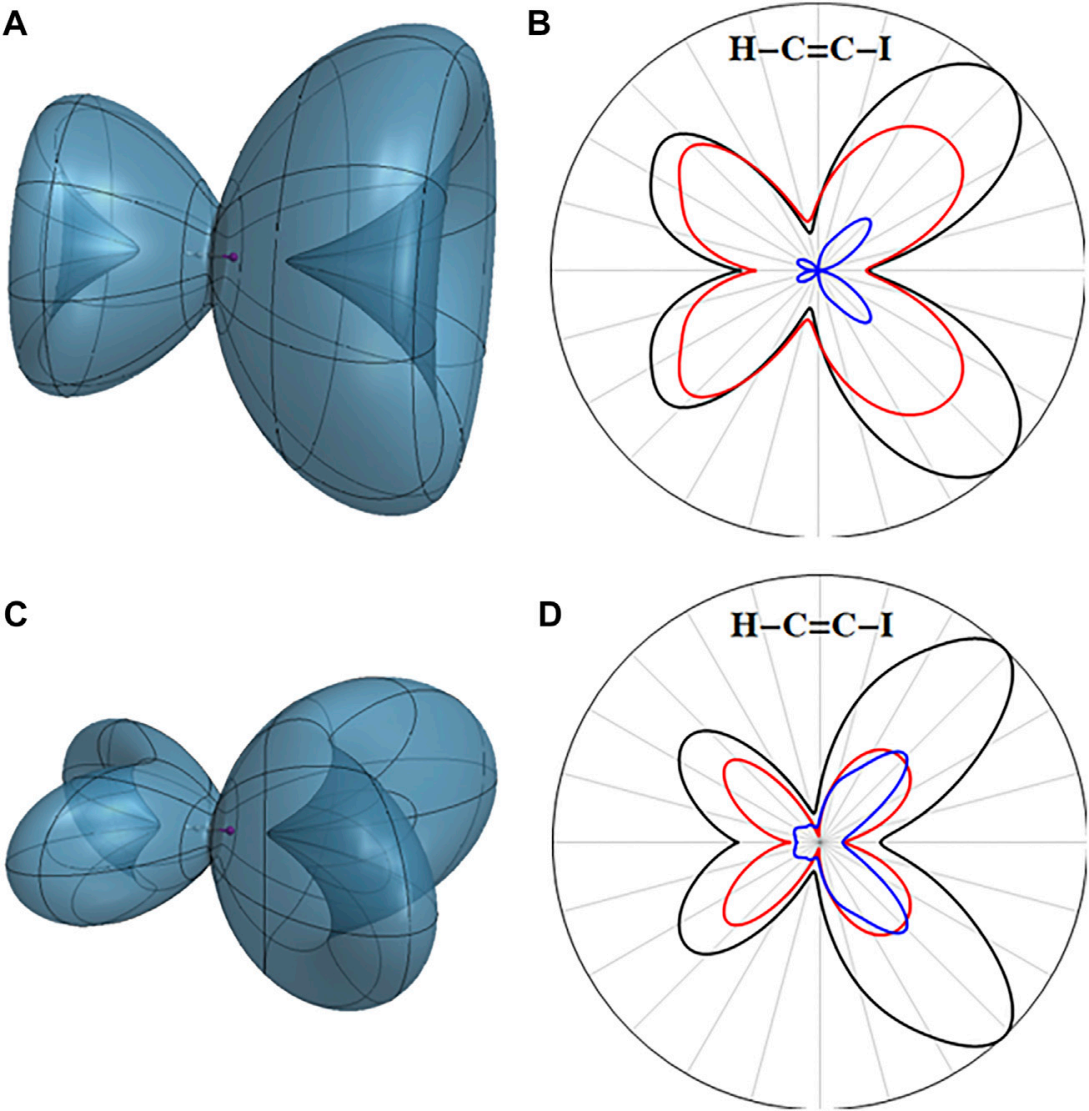
Three-dimensional angular dependence of the instantaneous ionization rate for the ground state of the HCCI cation in a static field of 0.055 au for **(A)** the X ( ${\bar{\pi }}_{+}^{*\;-1}$ ) cation and **(C)** the X ( ${\bar{\pi }}_{x}^{*\;-1}$ ) cation. Total ionization rates in the xz plane in black and contributions from ionizing the $\pi^{*}$ orbitals in red and from the π orbitals in blue for **(B)** the X ( ${\bar{\pi }}_{+}^{*\;-1}$ ) cation and **(D)** the X ( ${\bar{\pi }}_{x}^{*\;-1}$ ) cation.

The electron density of the field-free X cation is cylindrically symmetrical with a hole in one of the degenerate $\pi_{\pm }^{*}$ orbitals (Figure 3A). The ionization from the X cation to the dication is dominated by removing an electron from $\pi_{\mp }^{*}$ , the other orbital of the degenerate pair (red curve in Figure 3B); contributions from the lower lying $\pi_{\pm }$ orbitals are smaller (blue curve). For HCCI aligned with the *z* axis, sequential strong field ionization of HCCI in a static field, or by linear and circular pulses with the electric field in the xz plane will favor cations and dications with electron hole densities localized in the xz plane, *i.e.* loss of electrons from the $\pi_{x}$ and $\pi_{x}^{*}$ orbitals. The three-dimensional angular dependence of the ionization rate for the X ( ${\bar{\pi }}_{x}^{*\;-1}$ ) cation is shown in Figure 3C. The largest contribution comes from the ${\bar{\pi }}_{y}^{*}$ orbital. If only ionization in the xz plane is considered, removing an electron from the $\pi_{x}^{*}$ orbital makes the largest contribution, yielding a ( $\pi_{x}^{*\;-1}$ , ${\bar{\pi }}_{x}^{*\;-1}$ ) dication (red curve in Figure 3D). Ionization from the $\pi_{x}$ and ${\bar{\pi }}_{x}$ orbitals make somewhat smaller contributions (blue curve).

**FIGURE 4:**
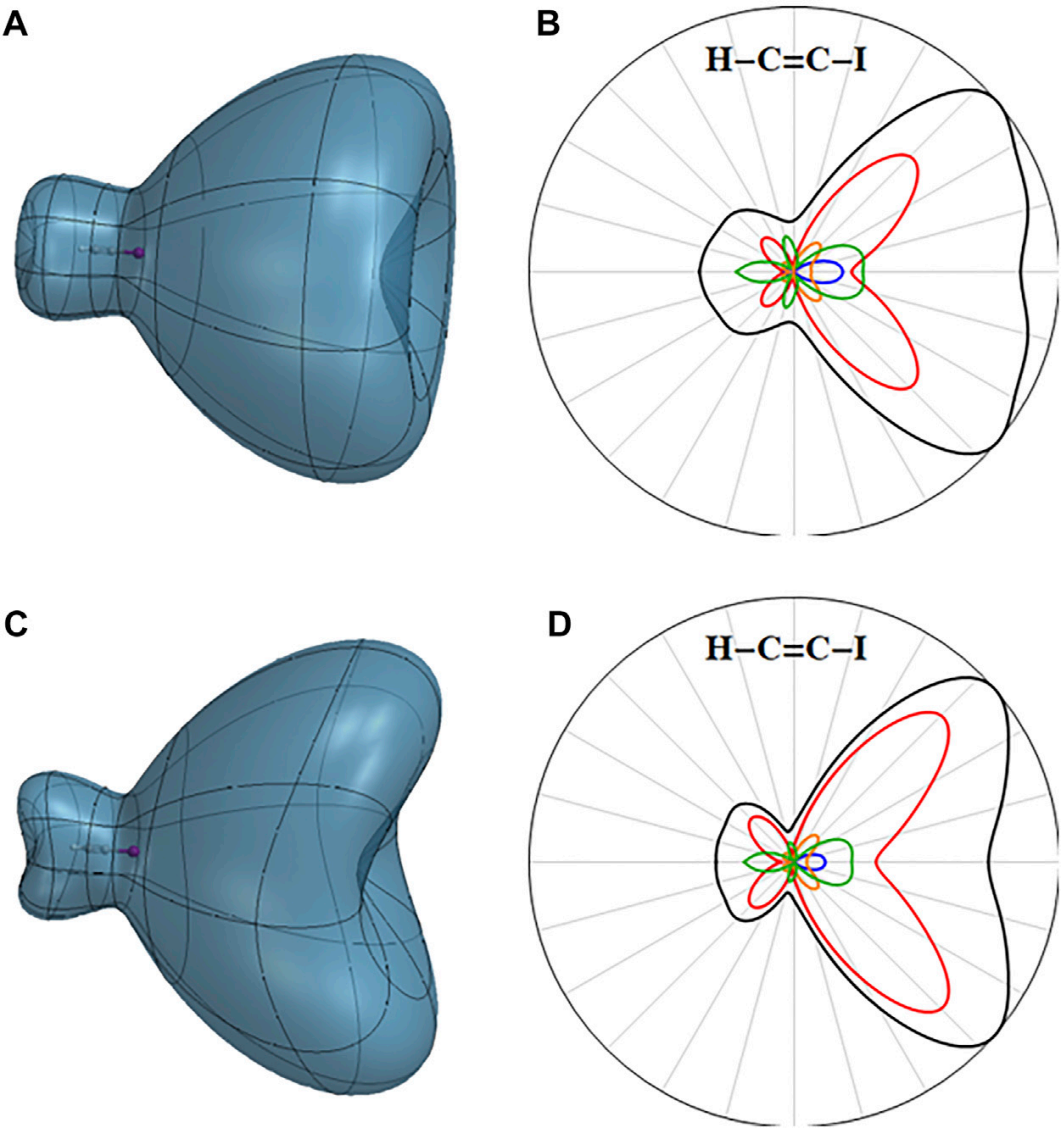
Three-dimensional angular dependence of the instantaneous ionization rate for the first excited state of the HCCI cation in a static field of 0.055 au for **(A)** the A ( ${\bar{\pi }}_{+}^{\;-1}$ ) cation and **(C)** the A ( ${\bar{\pi }}_{x}^{\;-1}$ ) cation. Total ionization rates in black and contributions from ionizing the $\pi^{*}$ and ${\bar{\pi }}^{*}$ orbitals in red (*i.e.* yielding the ( ${\bar{\pi }}^{-1}$ , $\pi^{*-1}$ ) and ( ${\bar{\pi }}^{-1}$ , ${\bar{\pi }}^{*-1}$ ) doubly ionized configurations), contributions yielding the ( $\pi^{*-1}$ , ${\bar{\pi }}_{y}^{\;-1}$ ), ( ${\bar{\pi }}^{*\;-1}$ , ${\bar{\pi }}_{y}^{-1}$ ), ( $\pi^{-1}$ , ${\bar{\pi }}_{y}^{*\;-1}$ ), and ( ${\bar{\pi }}^{-1}$ , ${\bar{\pi }}_{y}^{*\;-1}$ ) doubly ionized configurations in blue, the ( $\pi^{-1}$ , ${\bar{\pi }}_{x}^{*\;-1}$ ) configurations in orange and the ( $\pi^{*\;-1}$ , $\pi^{*-1}$ ), ( $\pi^{*-1}$ , ${\bar{\pi }}^{*-1}$ ), and ( ${\bar{\pi }}^{*-1}$ , ${\bar{\pi }}^{*-1}$ ) configurations in green for **(B)** the A ( ${\bar{\pi }}_{+}^{-1}$ ) cation and **(D)** the A ( ${\bar{\pi }}_{x}^{\;-1}$ ) cation.

The angular dependence of the ionization rate for the A state of the cation with a hole in the $\bar{\pi }$ orbital is shown in Figure 4 along with the orbital contributions. Because the A state is 2.2 eV higher than the X state, its ionization rate is considerably higher. As expected, the largest contribution to ionization comes from removing an electron from the $\pi^{*}$ orbitals (red curve in Figures 4B,D). Because of large transition dipole matrix elements between the π and $\pi^{*}$ orbitals (0.88 au), the component of the electric field aligned with the molecular axis mixes the X and A field-free states. As discussed above, the component from the X state ionizes mainly from $\pi^{*}$ orbitals, yielding a dication with 2 electrons removed from the $\pi^{*}$ orbitals (green curve in Figures 4B,D). There is also some ionization of the A ( ${\bar{\pi }}_{x}^{-1}$ ) cation along the molecular axis from the iodine end (blue curve). The partitioning of this ionization rate into orbital contributions indicates that it involves ionization from the $\pi_{y}$ and $\pi_{y}^{*}$ orbitals when the field is aligned with the molecular axis. This component becomes considerably larger for higher field strengths.

**FIGURE 5:**
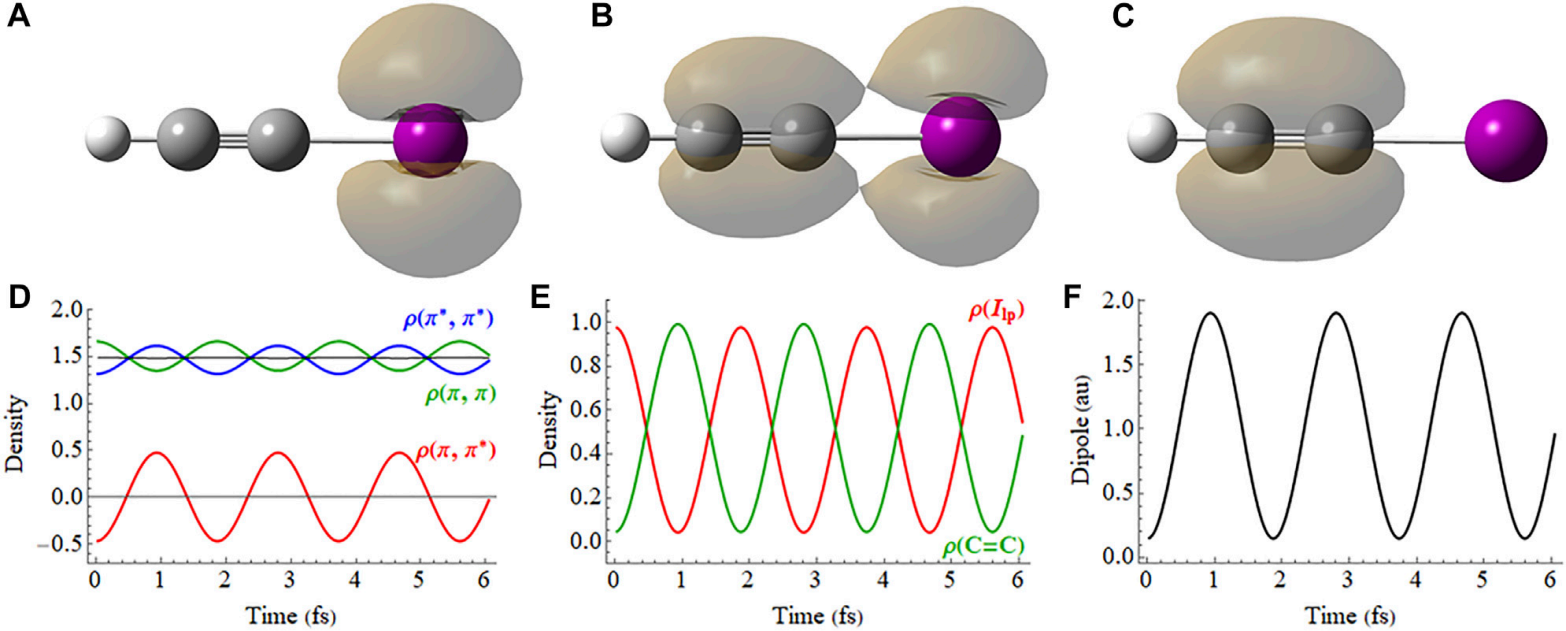
Field-free coherent superposition of the X ( ${\bar{\pi }}_{x}^{*\;-1}$ ) and A ( ${\bar{\pi }}_{x}^{\;-1}$ ) cations of HCCI. Hole density for **(A)** X + A, **(B)** X + *i* A and **(C)** X − A (a movie of the hole density is available in Supplementary Figure S1 of the Supporting Information). **(D)** Density matrix elements for the $\pi_{x}$ and $\pi_{x}^{*}$ orbitals. **(E)** Hole density for the C=C π orbital ( $\pi -\pi^{*}$ ) and the I_lp_ orbital ( $\pi +\pi^{*}$ ). **(F)** Z component of the dipole moment.

In the field-free case, the X ( ${\bar{\pi }}_{x}^{*\;-1}$ ) and A ( ${\bar{\pi }}_{x}^{\;-1}$ ) cations are stationary states, but a coherent superposition of these X and A cations results in a hole density that oscillates continuously between the iodine *π*-type lone pair and the CC *π* bond, as shown in Figures 5A–C (a movie is available in Supplementary Figure S1 of the Supporting Information). The density for the π and $\pi^{*}$ orbitals is shown in Figure 5D. The charge oscillation can be seen readily by examining the hole density for the C=C π orbital and the *π*-type I_lp_ orbital, plotted in Figure 5E. The charge oscillation can also be seen by monitoring the dipole moment, Figure 5F. The period of the oscillation, 1.87 fs, is determined by the 2.21 eV energy difference between the X and A states. The CISD-IP wavefunctions for the field-free cations involve a small amount of mixing between the pure ( ${\bar{\pi }}_{x}^{*\;-1}$ ) and ( ${\bar{\pi }}_{x}^{\;-1}$ ) configurations Ψ(X) = 0.95 ( ${\bar{\pi }}_{x}^{*\;-1}$ ) - 0.14 ( ${\bar{\pi }}_{x}^{\;-1}$ ) and Ψ(A) = 0.93 ( ${\bar{\pi }}_{x}^{\;-1}$ ) + 0.16 ( ${\bar{\pi }}_{x}^{*\;-1}$ ))

**FIGURE 6:**
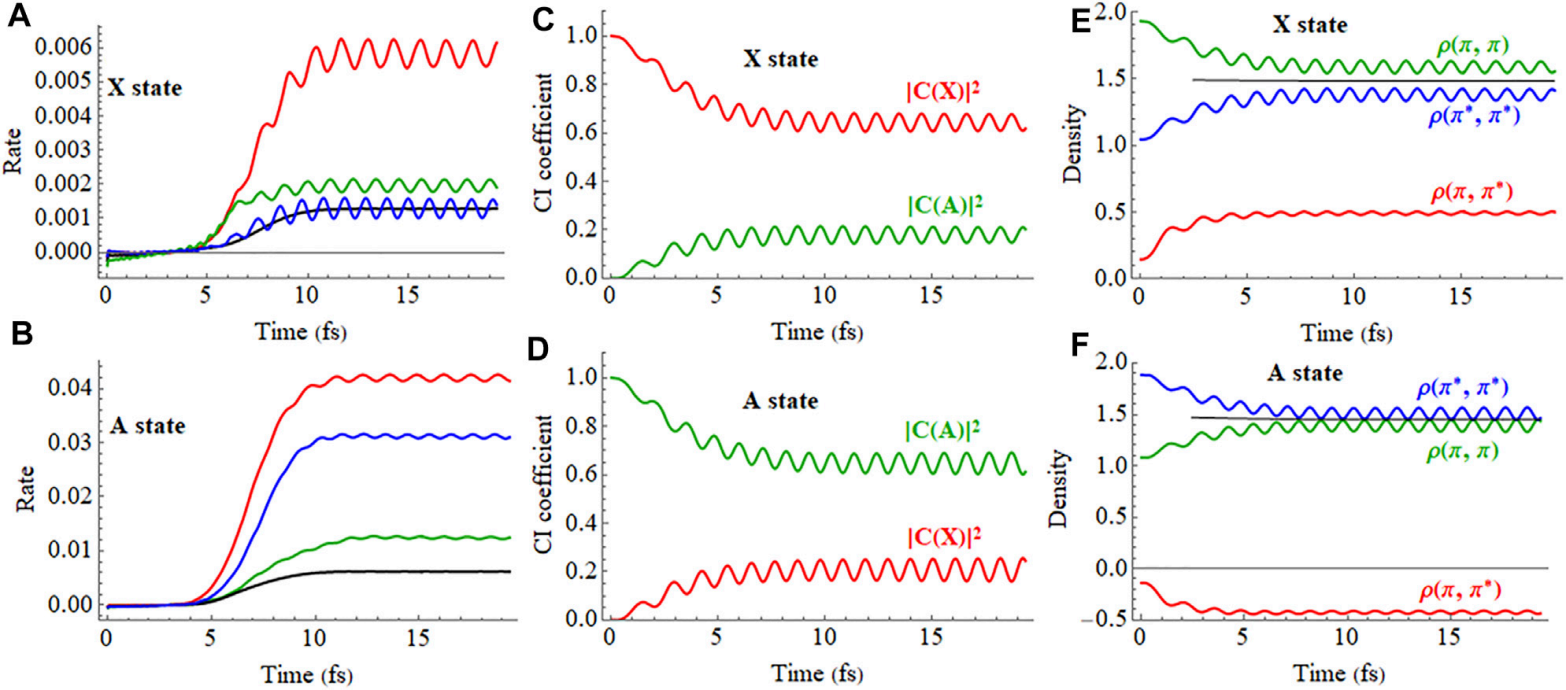
Ionization of the X ( ${\bar{\pi }}_{x}^{*\;-1}$ ) and A ( ${\bar{\pi }}_{x}^{\;-1}$ ) states by static electric field ramped up to 0.055 au. **(A,B)** The ionization rate for the field along the molecular axis and ionization from the iodine end (blue) and the hydrogen end (green), perpendicular to the molecular axis (black) and at 45° to the molecular axis from the iodine end (red). **(C,D)** CI coefficients for the X and A configurations (red and green, resp.) in the normalized wavefunction for the time-dependent X and A states, for the field along the molecular axis and ionization from the iodine end. **(E,F)** Density matrix elements for the $\pi_{x}$ and $\pi_{x}^{*}$ orbitals for the field along the molecular axis and ionization from the iodine end for the X and A states, respectively. A movie of the hole density for the X state is available in Supplementary Figure S2 of the Supporting Information.

In a finite static field, the field-free X and A states are generally no longer stationary. Figures 6A,B show the X and A states for a number of directions of a static field that is ramped up to a constant value of 0.055 au (a movie of the hole density for the X state is available in Supplementary Figure S2 of the Supporting Information). As expected, the ionization rate for the higher lying A state is considerably larger than for the X state. When the field has a component along the molecular axis, the X and A configurations interact through a large transition dipole matrix element. This mixing of the X and A states produces the oscillation of the ionization rate seen in Figures 6A,B. Because the X and A states have different dipole moments and polarizabilities, the finite field affects the energy difference between the two states and hence changes the oscillation frequency (*e.g.* compare the different directions shown Figure 6A and see Supplementary Figure S12 of Kraus et al., 2015). The square of the CI coefficients shown in Figures 6C,D are the populations of the field-free configurations (see Eq. 4). For the field along the molecular axis and ionization from the iodine end, the CI coefficient squared for the X configuration decreases from an initial value of 1 to an average of 0.64 and the coefficient squared for the A configuration increases from 0 to an average of 0.18; complementary behavior is seen for the A state. The density matrix elements in terms of the field-free π and $\pi^{*}$ molecular orbitals are shown in Figures 6E,F. The ρ(π, π) and $\rho \left(\pi^{*},\pi^{*}\right)$ matrix elements (*i.e.,* populations of the π and $\pi^{*}$ molecular orbitals) become nearly equal and average to a constant value of 1.5. For the X state, the off-diagonal $\rho \left(\pi ,\pi^{*}\right)$ rises to 0.5 and accounts for a shift of the electron density toward the CC *π* bond; the converse behavior is seen for the A state.

**FIGURE 7:**
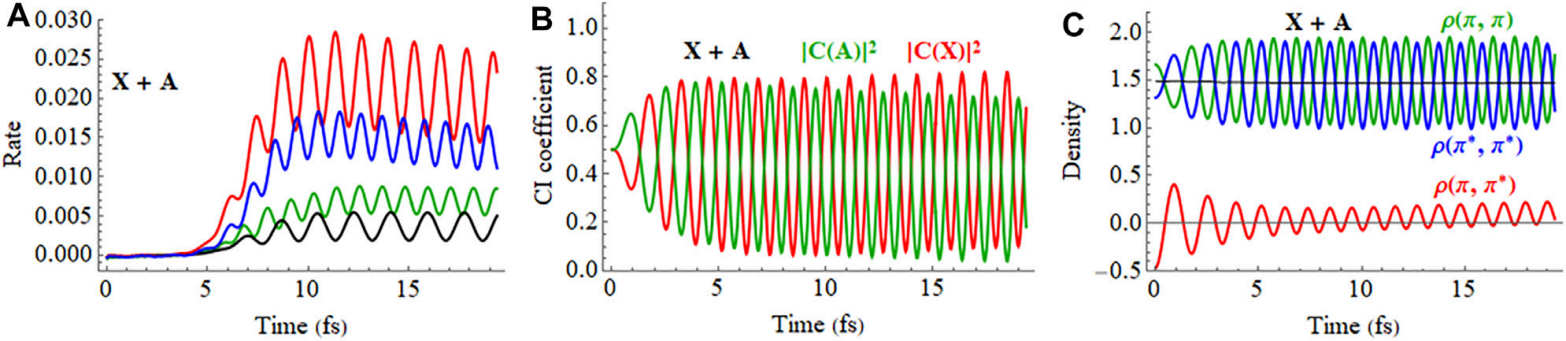
Ionization of the X + A ( ${\bar{\pi }}_{x}^{*\;-1}$ + ${\bar{\pi }}_{x}^{\;-1}$ ) coherent superposition by a static electric field ramped up to 0.055 au. **(A)** The ionization rate for the field along the molecular axis and ionization from the iodine end (blue) and the hydrogen end (green), perpendicular to the molecular axis (black) and at 45° to the molecular axis from the iodine end (red). **(B)** CI coefficients for the X and A configurations (red and green, resp.) in the normalized wavefunction for the X + A coherent superposition for the field along the molecular axis and ionization from the iodine end. **(C)** Density matrix elements for the $\pi_{x}$ and $\pi_{x}^{*}$ orbitals for the field along the molecular axis and ionization from the iodine end (a movie of the hole density is available in Supplementary Figure S2 of the Supporting Information).

The coherent superposition of X + A in a static field is shown in Figure 7 (a movie is available in Supplementary Figure S2 of the Supporting Information). Similar results were found for the X − A superposition. As expected from the field-free case, the electron hole moves between the iodine *π*-type lone pair and the CC *π* bond resulting in oscillations of the ionization rate for all field directions. Like the X and A states individually, the oscillation frequency for X + A ionization rate depends on the magnitude and direction of the field. After the field has reached a constant value, there is a noticeable decrease in the ionization rate from the iodine end of the molecule (red and blue curves in Figure 7A). The CI coefficients of the X and A configurations for ionization from the iodine end are plotted in Figure 7B. The X:A ratio changes from an initial value of 1:1 to an average value of 1:0.9 by the end of the simulation because the A state is ionized more rapidly than the X state. Since the ionization from the iodine end is 5–10 times higher for the A state than for the X state, (compare Figures 6A,B), the decrease in the relative population of the A state accounts for the decrease in the total ionization rate seen in Figure 7A. The density matrix elements are shown in Figure 7C. The average value of the off-diagonal $\rho \left(\pi ,\pi^{*}\right)$ matrix element increases from 0 to 0.12 by the end of the simulation as a result of the increasing fraction of the X configuration. The amplitude of the oscillations of $\rho \left(\pi ,\pi^{*}\right)$ decreases from ±0.5 at zero field strength to ±0.1 when the field reaches a constant value. When simulations of the coherent superposition of X + A are carried out in the absence of ionization (*e.g.,* no absorbing basis), the X:A ratio does not change and the average value of $\rho \left(\pi ,\pi^{*}\right)$ remains zero; this supports the conclusion that the decrease in the rate observed in Figure 7A is due to the more rapid ionization of the A configuration.

**FIGURE 8:**
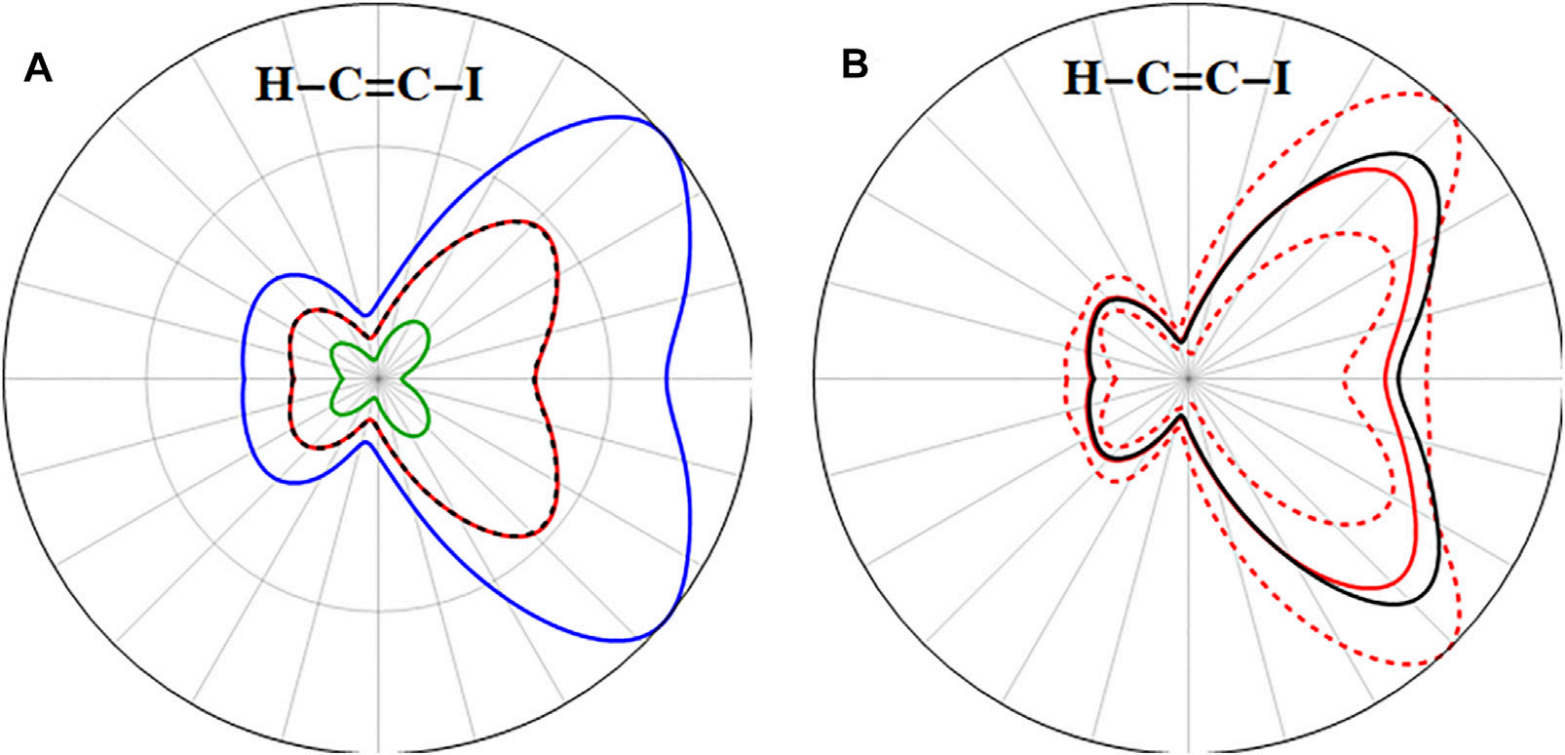
**(A)** Comparison of the ionization yield (decrease in $|\Psi {|}^{2}$ ) for the X + A coherent superposition (red) with the incoherent superposition of X and A (black dashed); the ionization yields for X and A states individually are in green and blue, respectively. **(B)** Comparison of the instantaneous ionization rate for the X + A coherent superposition (red) with the incoherent superposition of X and A (1:0.9 ratio, black) averaged over the last 2 fs of the simulation; maximum and minimum rates for X + A during the last 2 fs of the simulation shown as dashed red lines (same simulation parameters as in Figure 7).

The ionization yield (decrease in $|\Psi {|}^{2}$ ) for the X + A coherent superposition was found to be nearly equal to the ionization yield for the incoherent superposition of X and A, Figure 8A. Since the yield is the integration of the rate over the pulse, it is not very sensitive to fluctuations in the ionization rate caused by the coherent superposition of the states. The instantaneous rates for the superposition oscillate strongly and at different frequencies for different directions, as seen in Figure 7A. The angular shape of the minimum and maximum of the instantaneous rates for X + A (shown as dashed red lines in Figure 8B, obtained from the last 2 fs of the simulation) have characteristics of the shapes for ionizing the $\pi^{*}$ orbitals in the A state (see Figure 4D). The average of the ionization rates for the coherent superposition of X + A is in good agreement with the rates for the incoherent superposition of X and A, when accounting for the change in the relative populations of X and A at the end of the simulation.

A summary of the static field studies includes the following observations: 1) There is a strong directional dependence of the ionization rate and yield that is governed by the shape and energy of the orbitals being ionized. 2) The large transition dipole between the X and A states causes significant mixing between these states when the field has a component along the molecular axis. 3) The strong field alters the CI coefficients and electron density distribution for the X and A states. 4) For a coherent superposition of the X and A states, the field can alter both the magnitude and frequency of the charge oscillation. These observations can be used to help interpret the simulations of HCCI^+^ subject to short, intense probe pulses that are linearly and circularly polarized.

**FIGURE 9:**
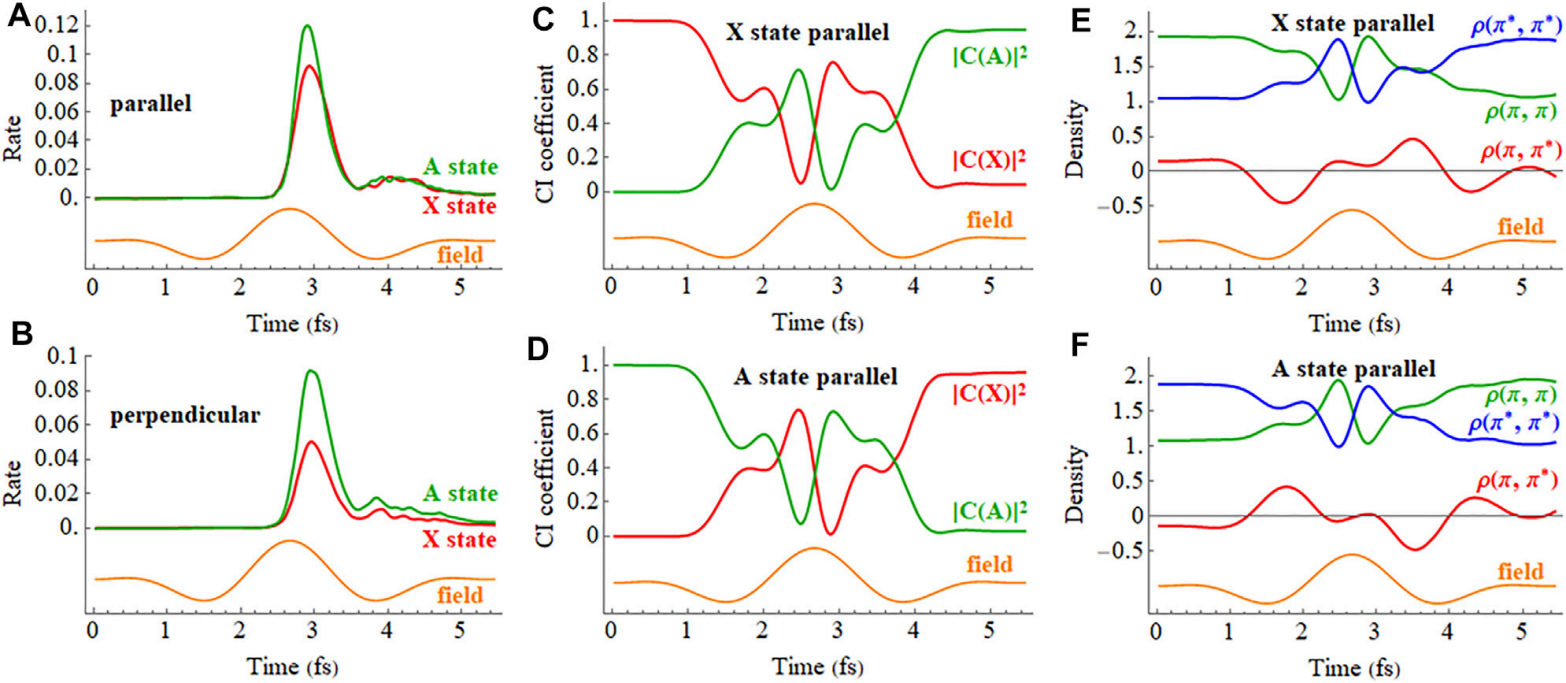
**(A,B)** Ionization rate for the X and A cations in a 2 cycle linearly polarized 800 nm pulse with *E*_max_ = 0.075 au aligned parallel and perpendicular to the molecular axis (X in red, A in green). **(C,D)** CI coefficients for the X and A configurations (red and green, resp.) in the normalized wavefunctions for the X ( ${\bar{\pi }}_{x}^{*\;-1}$ ) and A ( ${\bar{\pi }}_{x}^{\;-1}$ ) states with the pulse aligned parallel to the molecular axis with ionization mainly from the iodine. **(E,F)** Density matrix elements for the $\pi_{x}$ and $\pi_{x}^{*}$ orbitals in the X and A states for the pulse aligned with the molecular axis (the orange traces are proportional to the electric field). A movie of the hole density is available in Supplementary Figure S3 of the Supporting Information.

The ionization rates of the X and A cations in a 2 cycle linearly polarized pulse oriented parallel and perpendicular to the molecular axis are plotted in Figures 9A,B (a movie of the hole density for the X state is available in Supplementary Figure S3 of the Supporting Information). Like the static field case, a linearly polarized pulse along the molecular axis causes strong mixing between the X and A configurations in the time-dependent wavefunctions during the pulse, as can be seen in the plot of the CI coefficients and density matrix elements versus time in Figures 9C–F. The fact that the time dependence of the CI coefficients and density matrix elements of the A state are nearly mirror images of the X state indicates that HCCI^+^ is behaving like a two-state system, as was found in earlier simulations. (Kraus et al., 2015). The strong fields in these very short pulses cause both polarization along the molecular axis during the pulse and the population transfer between the X and A configurations by the end of the pulse. Figures 9C–F show that the mixing between the X and A configurations and oscillations in the electron density do not simply follow the period of driving the laser pulse (2.66 fs) but also contain components arising from the superposition of the X and A states (period of 1.87 fs for the field-free case). This is more evident for longer pulses (see Supplementary Figure S4 of the Supporting Information). Because the pulse produces a superposition of the X and A states, the oscillation in the $\rho \left(\pi ,\pi^{*}\right)$ density matrix element continues after the pulse. Polarization and population transfer are considerably less for other alignments of the pulse that have smaller components along the molecular axis. When the pulse is perpendicular to the molecular axis, there is no mixing between the X and A states because the transition dipole between these states is zero by symmetry for this direction.

**FIGURE 10:**
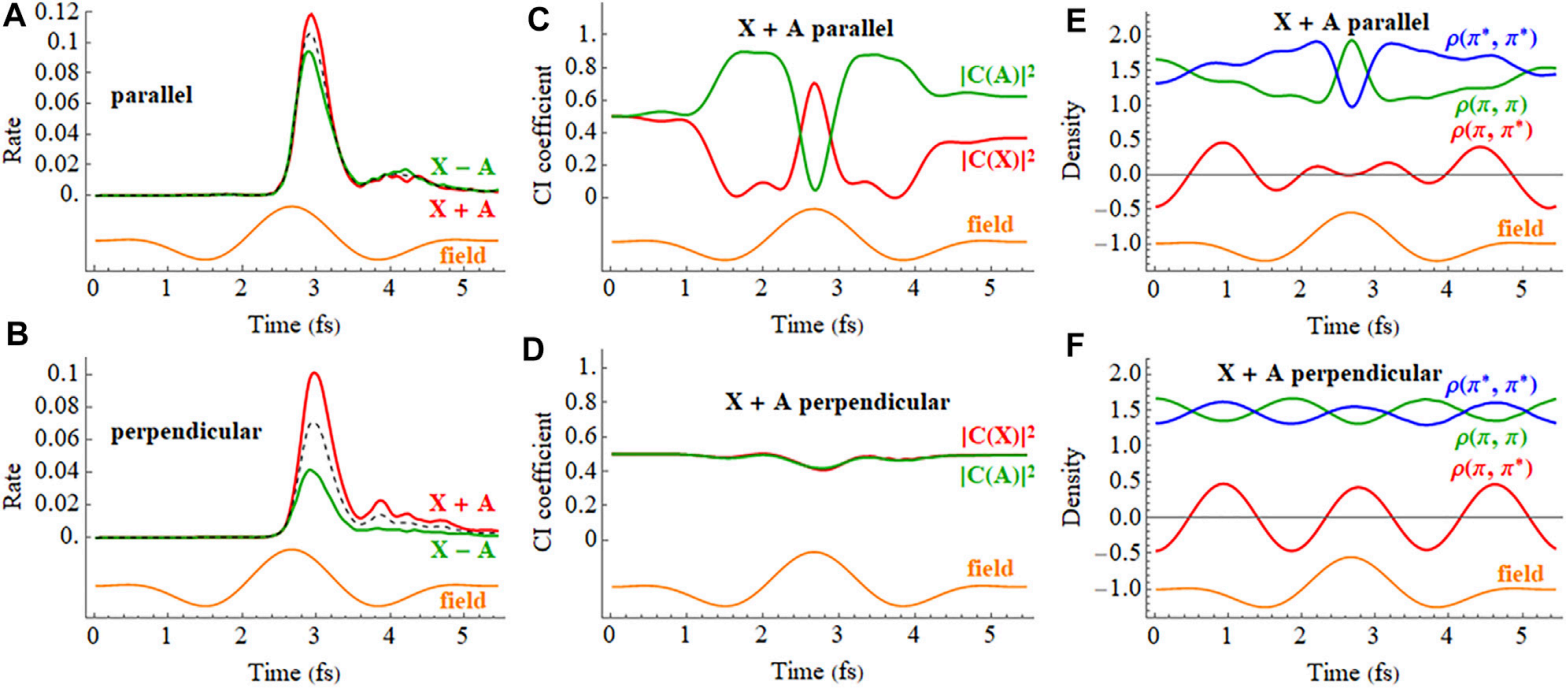
**(A,B)** Ionization rate for coherent X + A and X − A superpositions for a 2 cycle, 800 nm linearly polarized pulse with *E*_max_ = 0.075 au aligned parallel and perpendicular to the molecular axis (incoherent average of X and A in black dashed). **(C,D)** CI coefficients for the X and A configurations (red and green, resp.) in the normalized wavefunction for polarizations parallel and perpendicular to the molecular axis. **(E,F)** Density matrix elements for the $\pi_{x}$ and $\pi_{x}^{*}$ orbitals for polarizations parallel and perpendicular to the molecular axis (the orange traces are proportional to the electric field). A movie of the X + A hole density for a pulse parallel to the molecular axis is available in Supplementary Figure S3 of the Supporting Information.

The response of the X + A and X − A coherent superpositions in a 2 cycle, linearly polarized pulse is shown in Figure 10 (a movie for X + A is available in Supplementary Figure S3 of the Supporting Information). The ionization rates for the X + A and X − A deviate quite noticeably from the X and A states averaged incoherently. Furthermore, simulations starting from the X + A and X − A superpositions differ significantly, indicating there is also a strong dependence on the initial phase of the superposition. The CI coefficients of the X + A coherent superposition for a pulse aligned parallel to the molecular axis, Figure 10C, show a combination of two oscillations, reflecting the frequency of the laser pulse and the intrinsic frequency of charge oscillation of the superposition seen in the field-free case. Like the CI coefficients, the density matrix elements (Figure 10E) also show a combination of two frequencies during the pulse but return to the single frequency seen for the superposition in the absence of a field. By the end of the pulse, the CI coefficients for the X and A configurations differ from their initial values because the pulse has caused significant population transfer between the states, as was seen earlier in both experiment and simulations. (Kraus et al., 2015). Calculations in the present study show that the nature of this population transfer depends not only on the initial phase of the superposition, but also on the length of the pulse, the carrier envelope phase (CEP), the field strength, and the pulse orientation. For example, a pulse perpendicular to the molecular axis does not cause any interaction between the X and A states (Figure 10D). The small response in the CI coefficients reflects the polarization perpendicular to the molecular axis through interactions with higher lying states. Because the perpendicular field does not affect the X + A superposition, the oscillations of the density matrix elements (Figure 10F) and the dipole moment (not shown) are essentially the same as the field-free case (Figure 5).

**FIGURE 11:**
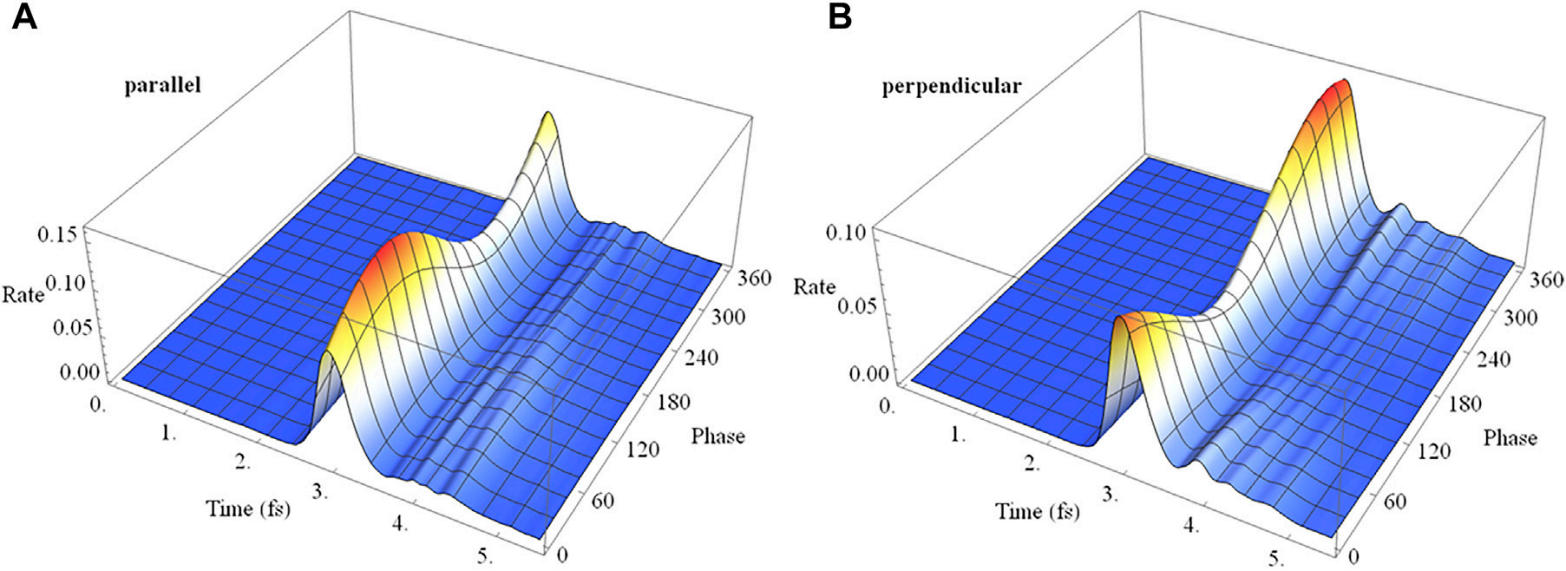
Ionization rate as a function of time and the initial phase of the superposition (X + e^i*ϕ*^ A) for a 2 cycle linearly polarized 800 nm pulse with *E*_max_ = 0.075 au for polarizations **(A)** parallel and **(B)** perpendicular to the molecular axis.

In a pump-probe experiment, the initial ionization of neutral HCCI by a pump pulse can produce a coherent superposition of the X and A cations. In the time delay between the pump and probe pulses, the phase of the superposition evolves with a field-free period of 1.87 fs. Thus, varying the pump-probe delay is equivalent to varying the initial phase of the superposition at the beginning of the probe pulse. Figure 11 shows the time dependence of the ionization rate and as a function of the phase of the superposition (X + e^i*ϕ*^ A) for polarizations parallel and perpendicular to the molecular axis. The ionization rates show clear maxima and minima with the variation of the initial phase (equivalent to the variation of the pump-probe time delay). The total ionization yield also shows corresponding maxima and minima with the variation of the initial phase. For a pulse perpendicular to the molecular axis (Figure 11B), the electron density migrates at the field-free rate and the maximum in the ionization rate occurs when the electron density on iodine is highest at the peak in the field. For a pulse parallel to the molecular axis (Figure 11A), the density starts to migrate at the field-free rate but is increasingly driven by the laser field as the intensity grows. As a result, the peak in the ionization rate is shifted to a different initial value of the superposition phase.

**FIGURE 12:**
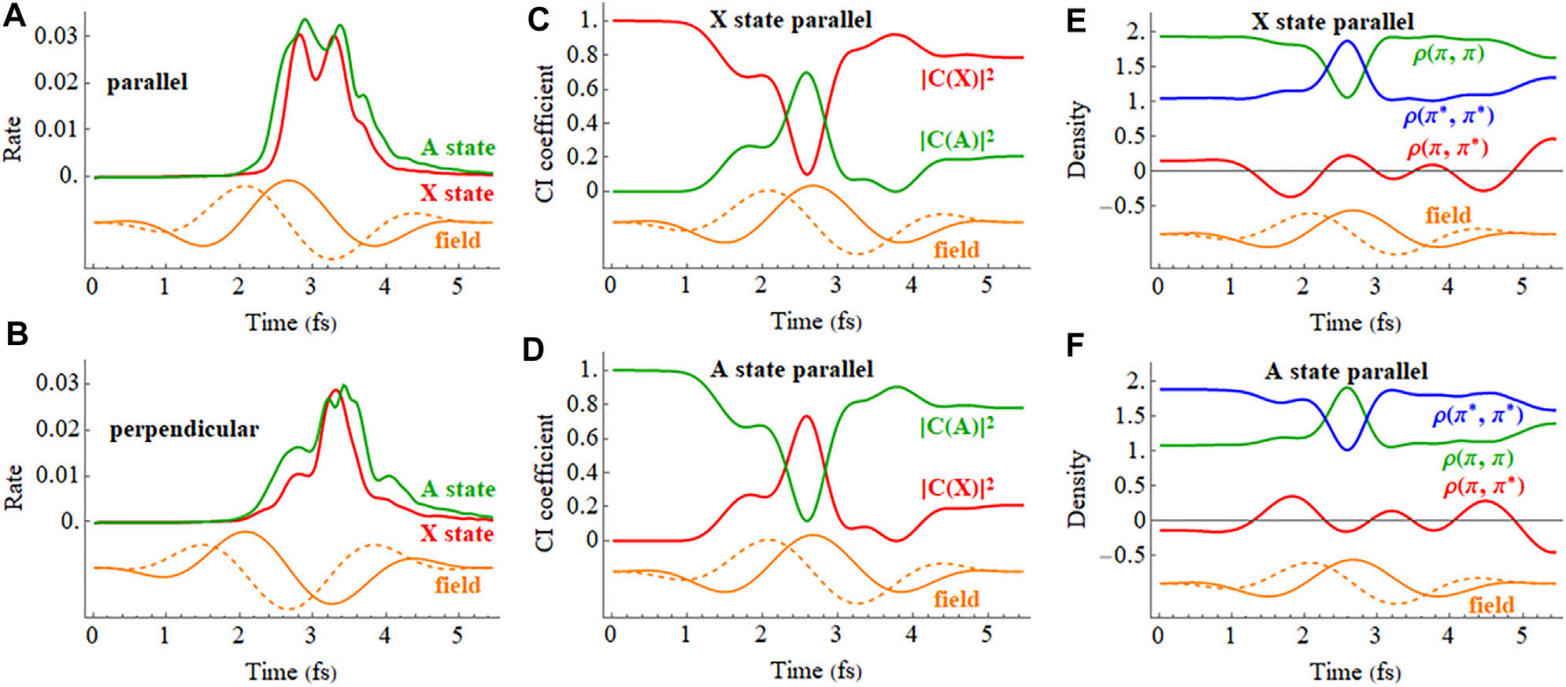
**(A,B)** Ionization rate for the X and A cations in a 2 cycle circularly polarized 800 nm pulse with *E*_max_ = 0.0566 au with the carrier envelope maximum parallel and perpendicular to the molecular axis (X in red, A in green). **(C,D)** CI coefficients for the X and A configurations (red and green, resp.) in the normalized wavefunctions for the X ( ${\bar{\pi }}_{x}^{*\;-1}$ ) and A ( ${\bar{\pi }}_{x}^{\;-1}$ ) states with the carrier envelope maximum parallel and perpendicular to the molecular axis. **(E,F)** Density matrix elements for the $\pi_{x}$ and $\pi_{x}^{*}$ orbitals in the X and A states with the carrier envelope maximum parallel to the molecular axis (the orange traces are proportional to the electric field). A movie of the hole density is available in Supplementary Figure S5 of the Supporting Information.

**FIGURE 13:**
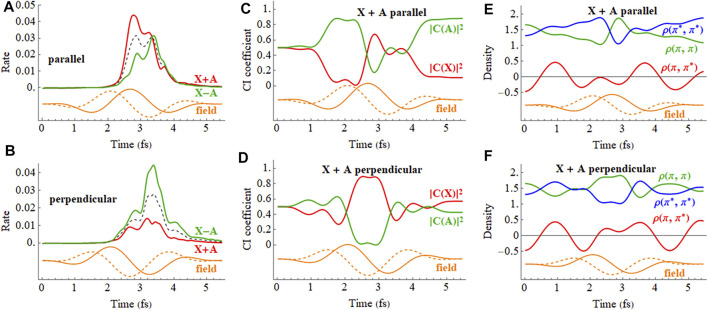
**(A,B)** Ionization rate for X + A and X − A coherent superpositions for a 2 cycle, 800 nm circularly polarized pulse with *E*_max_ = 0.0566 au with the carrier envelope maximum parallel and perpendicular to the molecular axis (incoherent average of X and A in black dashed). **(C,D)** CI coefficients for the X and A configurations (red and green, resp.) in the normalized wavefunction for X + A with the carrier envelope maximum parallel and perpendicular to the molecular axis. **(E,F)** Density matrix elements for the $\pi_{x}$ and $\pi_{x}^{*}$ orbitals for X + A (the orange traces are proportional to the electric field). A movie of the hole density is available in Supplementary Figure S3 of the Supporting Information.

The ionization rates for the X and A cations by a 2 cycle circularly polarized pulse are shown in Figure 12. The field rotates in the xz plane and the CEP is chosen so that the maximum in the field is either parallel to the molecular axis (*z* axis) with ionization from the iodine end or perpendicular to the molecular axis with the field rotating toward the iodine. Figures 12C,D indicate that the mixing between the X and A configurations is largest mid-pulse when the field is aligned with the molecular axis. There is some population transferred between the X and A configurations by the end of the pulse. The ionization rates for a coherent superposition of the X and A states in a circularly polarized pulse are plotted in Figure 13. Similar to a linear pulse, there is a significant difference in the peak ionization rate for the X + A and X − A superpositions. The CI coefficients and the density matrix elements show a combination of two oscillations, reflecting the frequency of the laser pulse and the intrinsic frequency of charge oscillation of the superposition.

**FIGURE 14:**
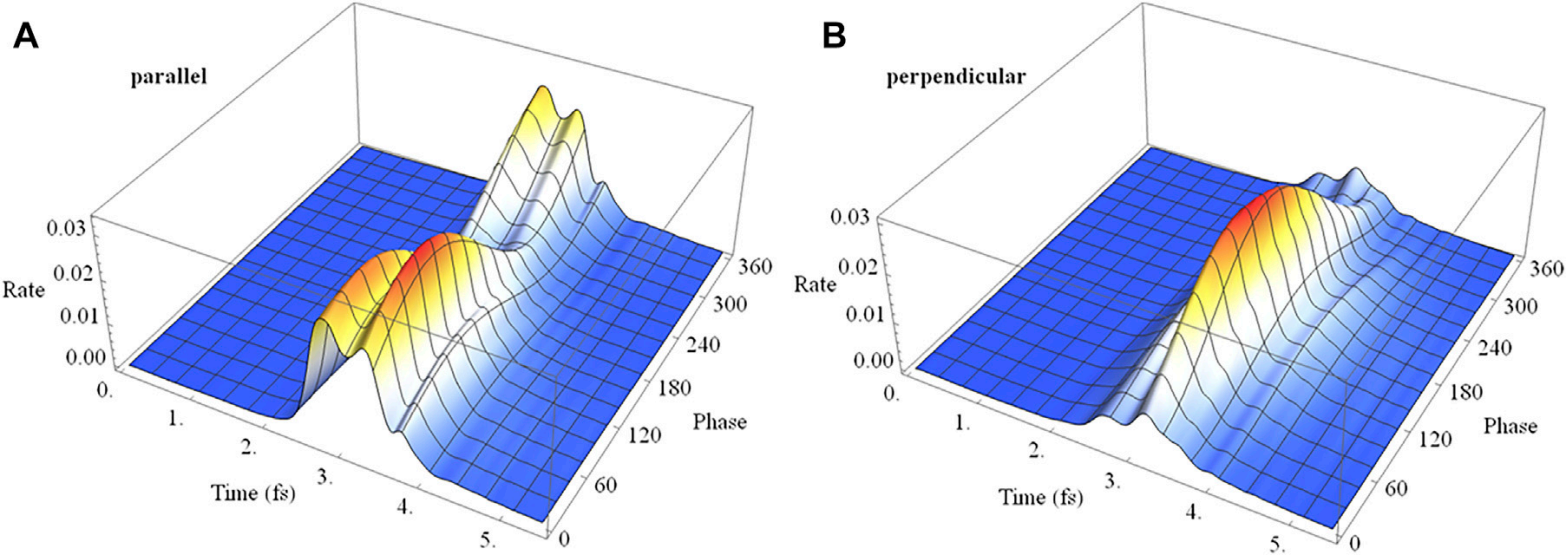
Ionization rate as a function of time and the initial phase of the superposition (X + e^i*ϕ*^ A) for 2 cycle circularly polarized 800 nm pulse with *E*_max_ = 0.0530 au with the carrier envelope maximum **(A)** parallel and **(B)** perpendicular to the molecular axis.

Analogous to the linear case, ionization of a coherent superposition of the X and A states of HCCI^+^ with a 2 cycle circularly polarized pulse varies with the initial phase of the superposition and with the carrier envelop phase, as shown in Figure 14. The rotating electric field drives the electron dynamics in a more complicated fashion than for a linearly polarized pulse. The variation can be understood with the help of the angular dependence of ionization in a static field discussed earlier. For the chosen parameters of the circularly polarized pulse, the electric field rotates in a clockwise direction for Figures 3D, 4D. When the maximum in the carrier envelope phase is parallel to the molecular axis and ionization is from the iodine end, the rate is a maximum at rotations of the field 45° before and after the molecular axis. This accounts for the two ridges seen in Figure 14A. The location of the maximum rate with respect to the initial superposition phase is similar to the linear case, Figure 11A, and depends on the interaction between the intrinsic charge migration dynamics and the driving laser field. When the maximum in the carrier envelope phase is perpendicular to the molecular axis and the field is rotating toward the iodine, the ionization rate has only one primary ridge. This occurs approximately 45° after the maximum when the field is aligned with the iodine π-type lone pair orbital (see Figures 3D, 4D). Because the electric field for the parallel and perpendicular cases affects the migration of the electron density differently, the maximum in the ionization rate occurs at different values for the initial superposition phases (corresponding to different pump-probe delay times).

## Summary

The electron dynamics and strong field ionization of HCCI neutral and cations in intense laser fields have been simulated using time-dependent configuration interaction with a complex absorbing potential. Ionization of neutral HCCI occurs from the $\pi^{*}$ and π orbitals, producing the X and A states of the cation, respectively. Use of a static field reveals the 3-dimensional angular dependence of strong field ionization and the orbitals involved in producing the cations and dications. Ionization of the neutral by a pump pulse can produce a coherent superposition of the X and A cations which results in charge oscillation between the CC *π* orbital and the iodine *π*-type lone pair. This migration can be monitored by ionizing with ultrashort probe pulses. Under field-free conditions, the frequency of charge oscillation is determined by the energy separation of the X and A states. However, the oscillation of the electron density is affected by the subsequent strong field ionization of the cations to dications by intense probe pulses. For fields along the molecular axis, strong field ionization of the individual X and A states causes extensive mixing between the X and A configurations resulting in charge oscillation between the CC *π* orbital and the iodine *π*-type lone pair during the pulse. For a coherent superposition, the oscillation of the charge shows two frequencies–the driving frequency of the laser field and the intrinsic frequency due to the energy separation between the X and A states. Strong field ionizations with linear and circularly polarized pulses show marked differences in the ionization rates that depend on the initial phase of the superposition, the polarization direction and the carrier envelope phase. Scanning the initial phase of the superposition is analogous to changing the delay between the pump and probe pulses. The charge oscillation resulting from the coherent superposition of the X and A states is seen in the variation in the ionization yield as a function of the initial phase of the superposition. Linearly polarized probe pulses perpendicular to the molecular axis do not affect the superposition and the charge oscillation frequency is the same as the field-free case. For linearly polarized pulses parallel to the molecular axis and for circularly polarized pulses, there is strong polarization along the molecular axis and the dynamics of the charge oscillation depends on the driving laser field as well as the intrinsic frequency resulting from the coherent superposition.

Finally, we briefly comment on the feasibility of experimental implementation of using strong field ionization to probe electronic wave packets. The proposed two-cycle laser pulses are indeed practical as single- or sub-cycle (FWHM) pulses have been produced with either a hollow-core fiber compressor or pulse synthesizer. (Hassan et al., 2016; Liang et al., 2017; Timmers et al., 2017). Our calculations also utilized pulses with fixed carrier-envelop phases. A technique for measuring the absolute CEPs of ultrashort pulses has been recently developed. (Debrah et al., 2019). While the ionization yield variation detection might require orientation of the molecules in space, it is rather straightforward to achieve this with an electron-ion coincidence method (Winney et al., 2018) in which the recoil momentum of the dissociated ion can be used to measure the orientation of molecules. Therefore, all necessary techniques that are needed to implement the current study are now available. The advantage of the proposed approach lies in its superb time resolution as well as an extended delay range, which will be crucial for studying electron wave packet decoherence and recoherence dynamics.

## Data Availability

The raw data supporting the conclusion of this article will be made available by the authors, without undue reservation.
