# Supplementary material for: Ionization of HCCI Neutral and Cations by Strong Laser Fields Simulated With Time Dependent Configuration Interaction
Source: Front Chem. 2022 Apr 25;10:866137. doi: 10.3389/fchem.2022.866137 (PMC9081608; doi:10.3389/fchem.2022.866137)
Supplement: Supplementary file 1 [file Presentation1.PPTX]

## Slide 1
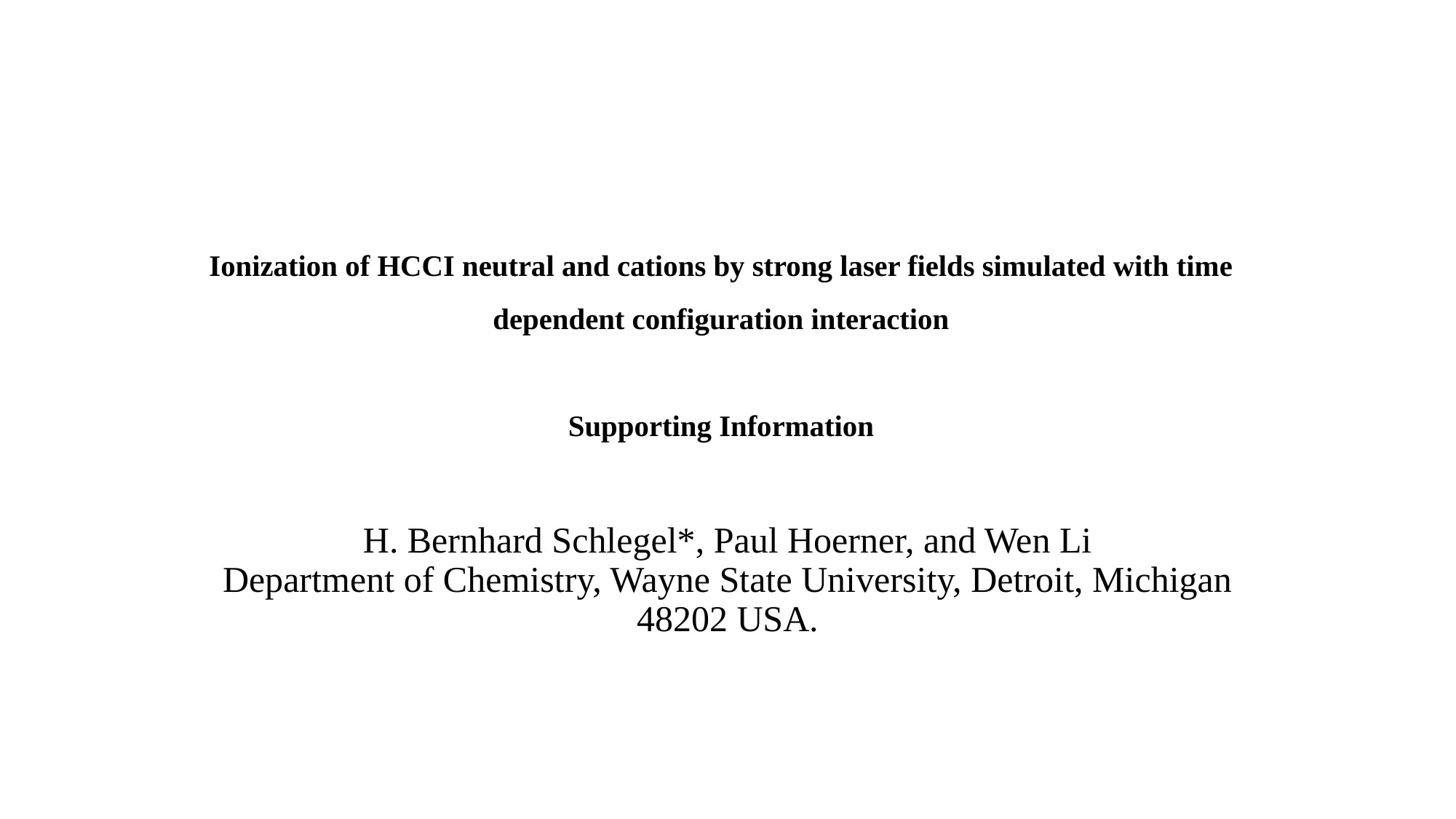

# Ionization of HCCI neutral and cations by strong laser fields simulated with time dependent configuration interaction Supporting Information
H. Bernhard Schlegel*, Paul Hoerner, and Wen Li
Department of Chemistry, Wayne State University, Detroit, Michigan 48202 USA.

## Slide 2
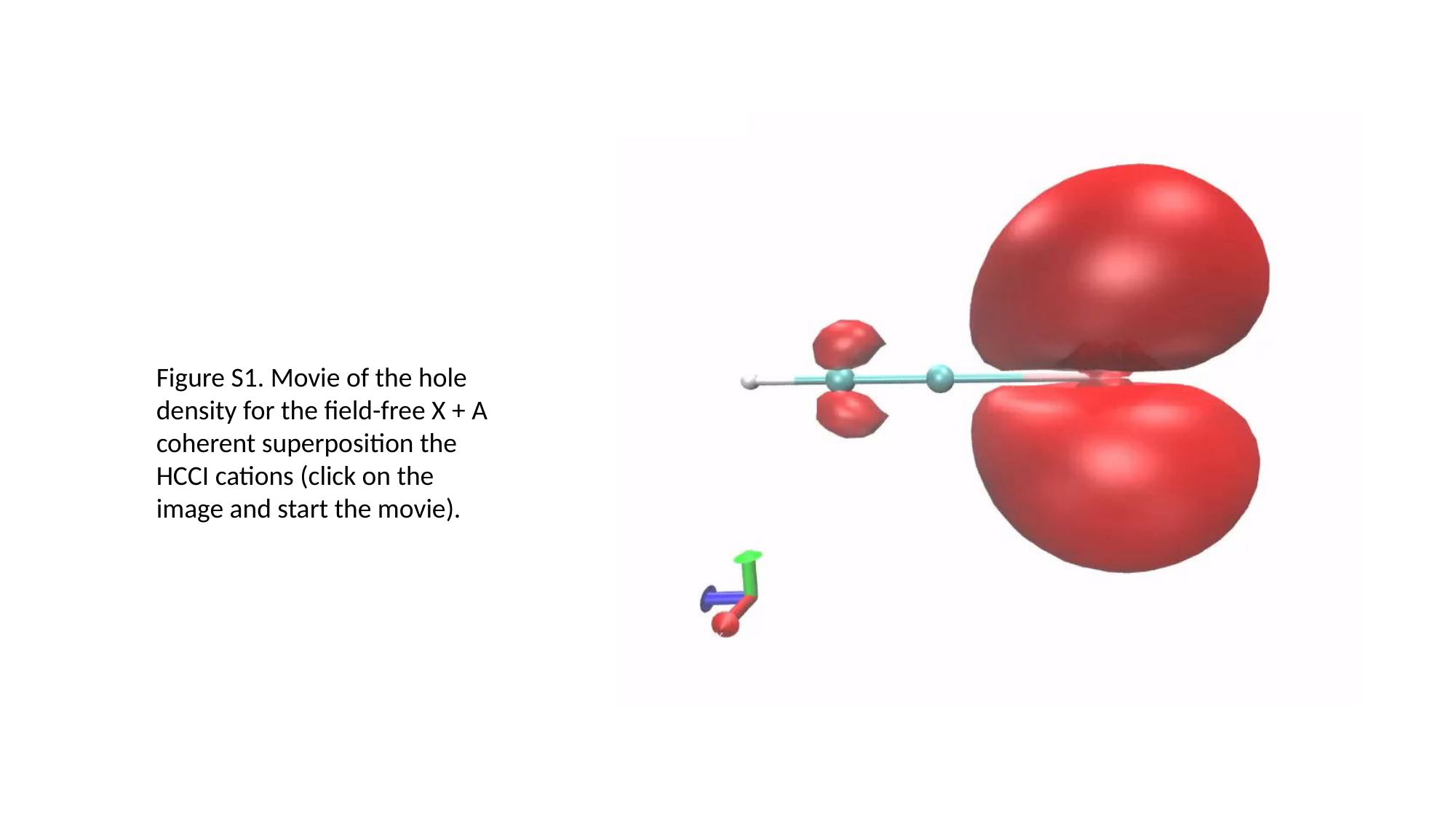

Figure S1. Movie of the hole density for the field-free X + A coherent superposition the HCCI cations (click on the image and start the movie).

## Slide 3
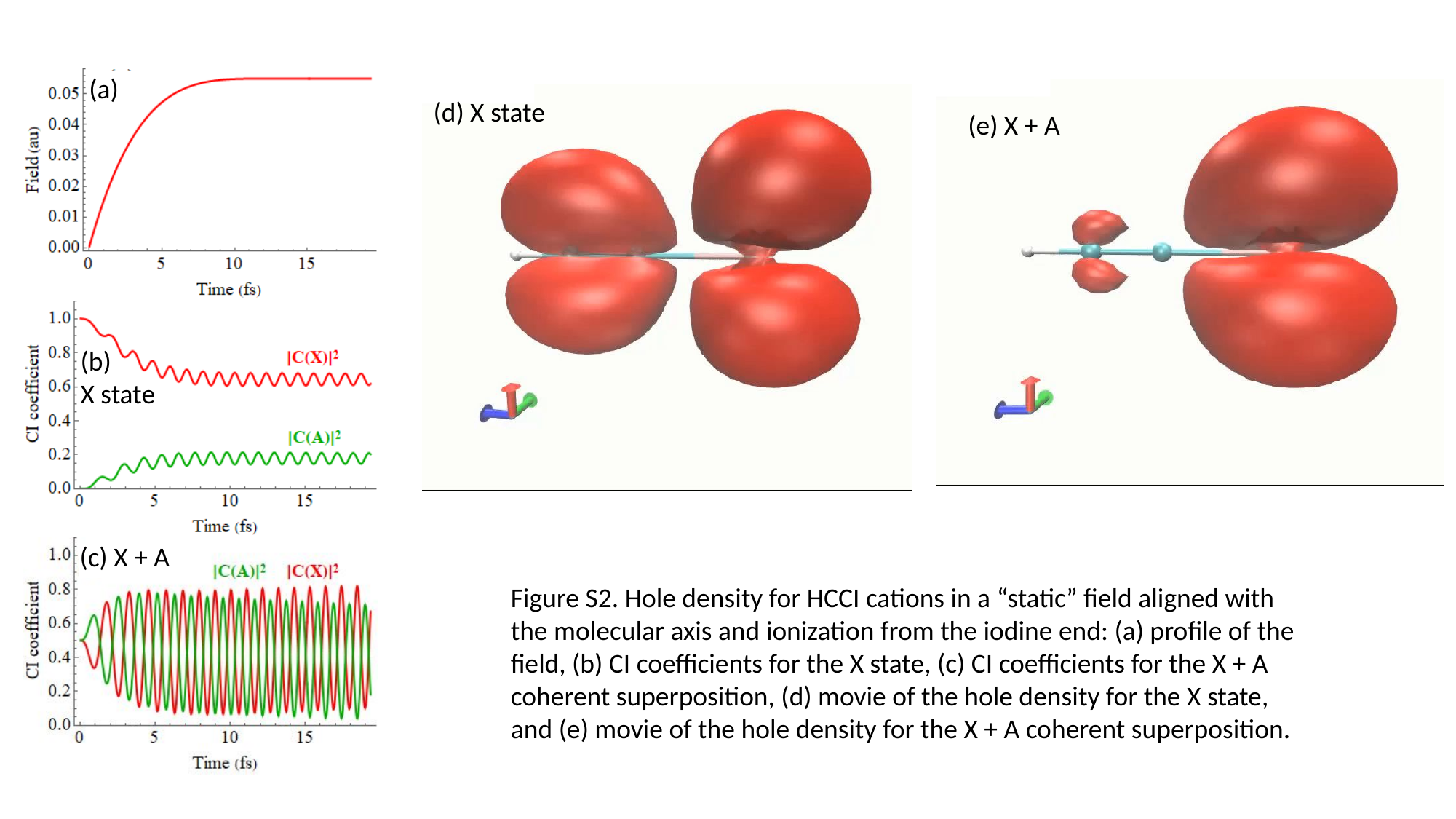

(a)
(b)
X state
(c) X + A
(e) X + A
(d) X state
Figure S2. Hole density for HCCI cations in a “static” field aligned with the molecular axis and ionization from the iodine end: (a) profile of the field, (b) CI coefficients for the X state, (c) CI coefficients for the X + A coherent superposition, (d) movie of the hole density for the X state, and (e) movie of the hole density for the X + A coherent superposition.

## Slide 4
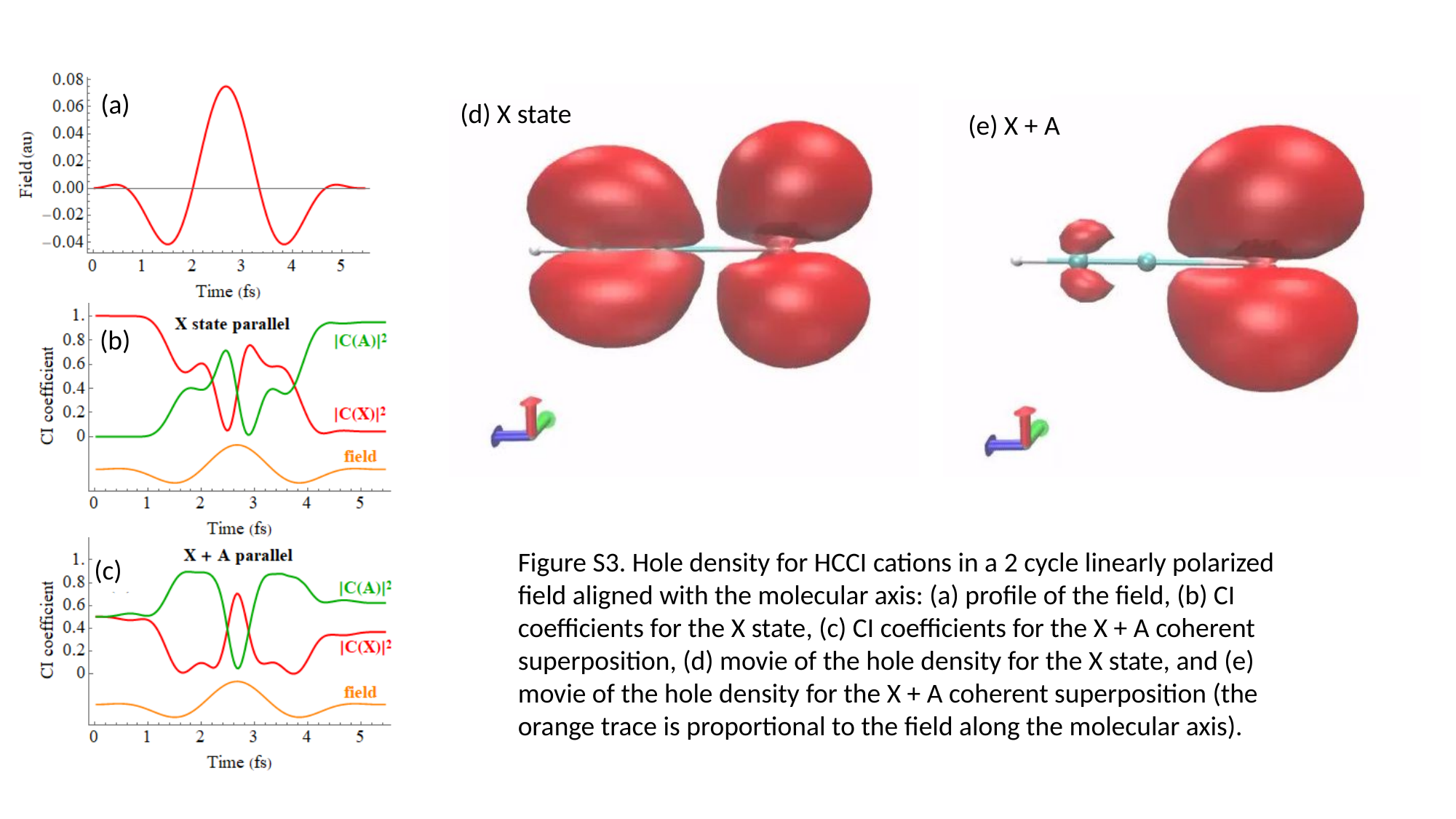

(d) X state
(e) X + A
(a)
(b)
Figure S3. Hole density for HCCI cations in a 2 cycle linearly polarized field aligned with the molecular axis: (a) profile of the field, (b) CI coefficients for the X state, (c) CI coefficients for the X + A coherent superposition, (d) movie of the hole density for the X state, and (e) movie of the hole density for the X + A coherent superposition (the orange trace is proportional to the field along the molecular axis).
(c)

## Slide 5
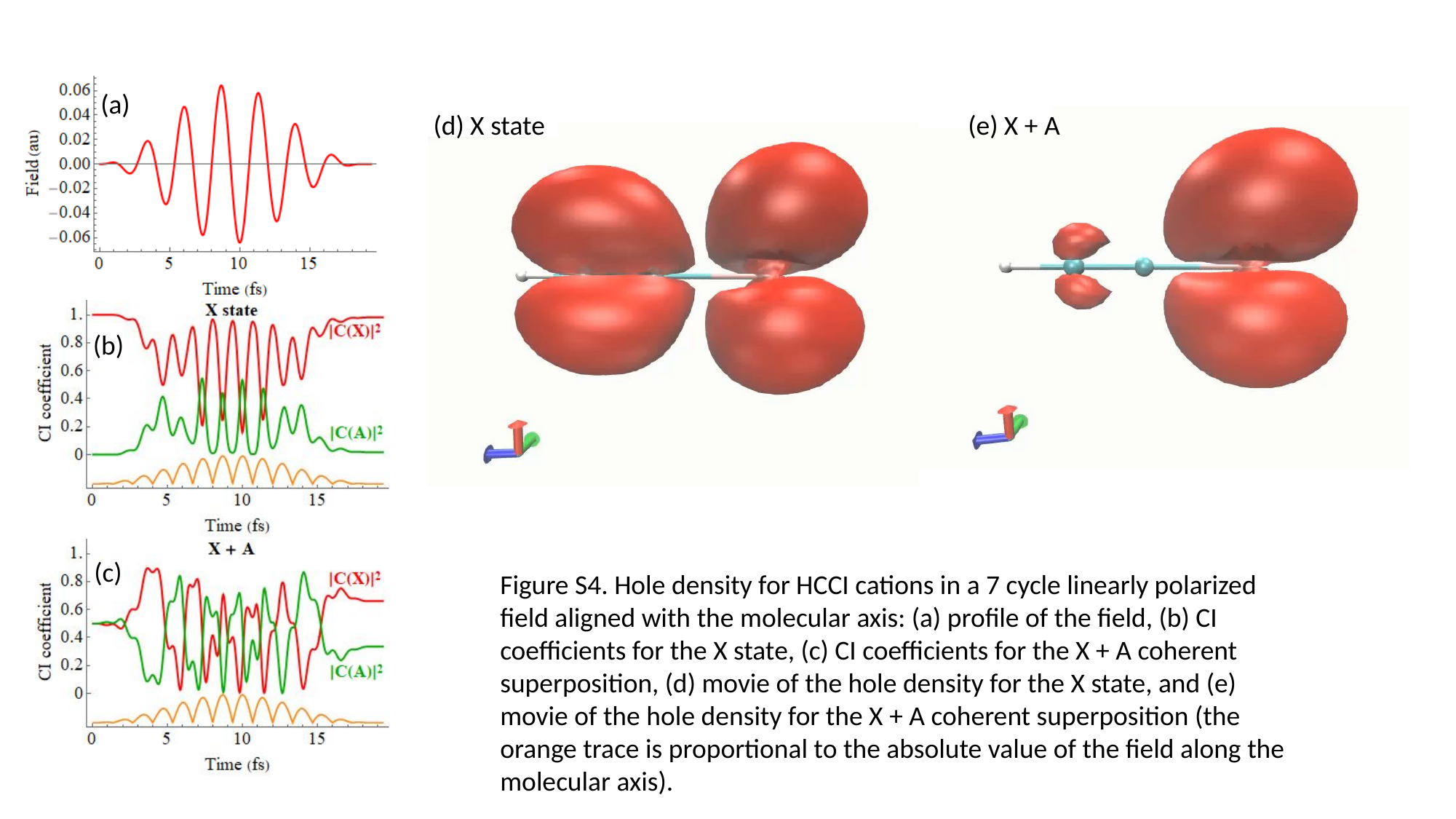

(a)
(b)
(c)
(e) X + A
(d) X state
Figure S4. Hole density for HCCI cations in a 7 cycle linearly polarized field aligned with the molecular axis: (a) profile of the field, (b) CI coefficients for the X state, (c) CI coefficients for the X + A coherent superposition, (d) movie of the hole density for the X state, and (e) movie of the hole density for the X + A coherent superposition (the orange trace is proportional to the absolute value of the field along the molecular axis).

## Slide 6
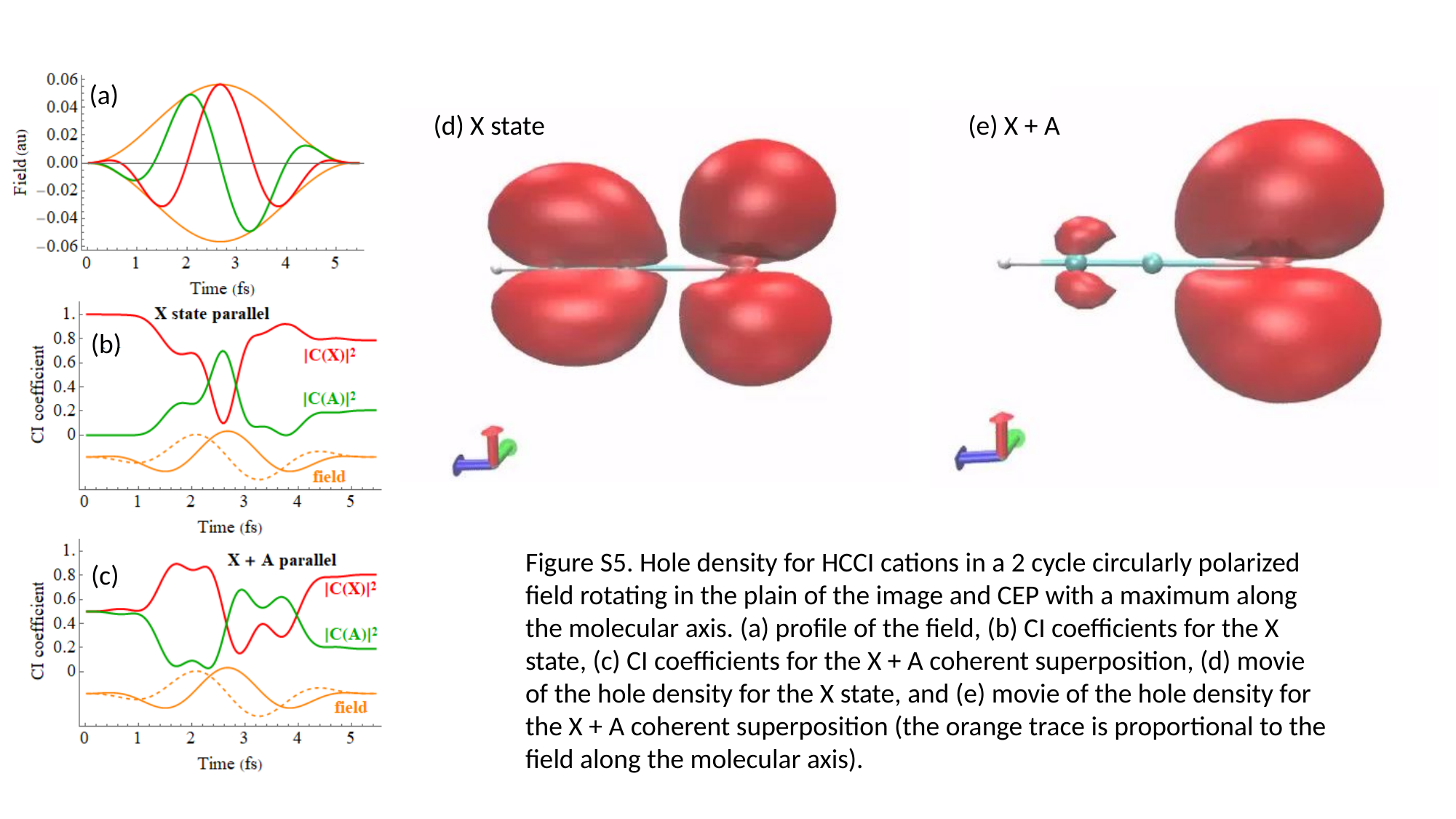

(d) X state
(a)
(e) X + A
(b)
Figure S5. Hole density for HCCI cations in a 2 cycle circularly polarized field rotating in the plain of the image and CEP with a maximum along the molecular axis. (a) profile of the field, (b) CI coefficients for the X state, (c) CI coefficients for the X + A coherent superposition, (d) movie of the hole density for the X state, and (e) movie of the hole density for the X + A coherent superposition (the orange trace is proportional to the field along the molecular axis).
(c)

## Slide 7
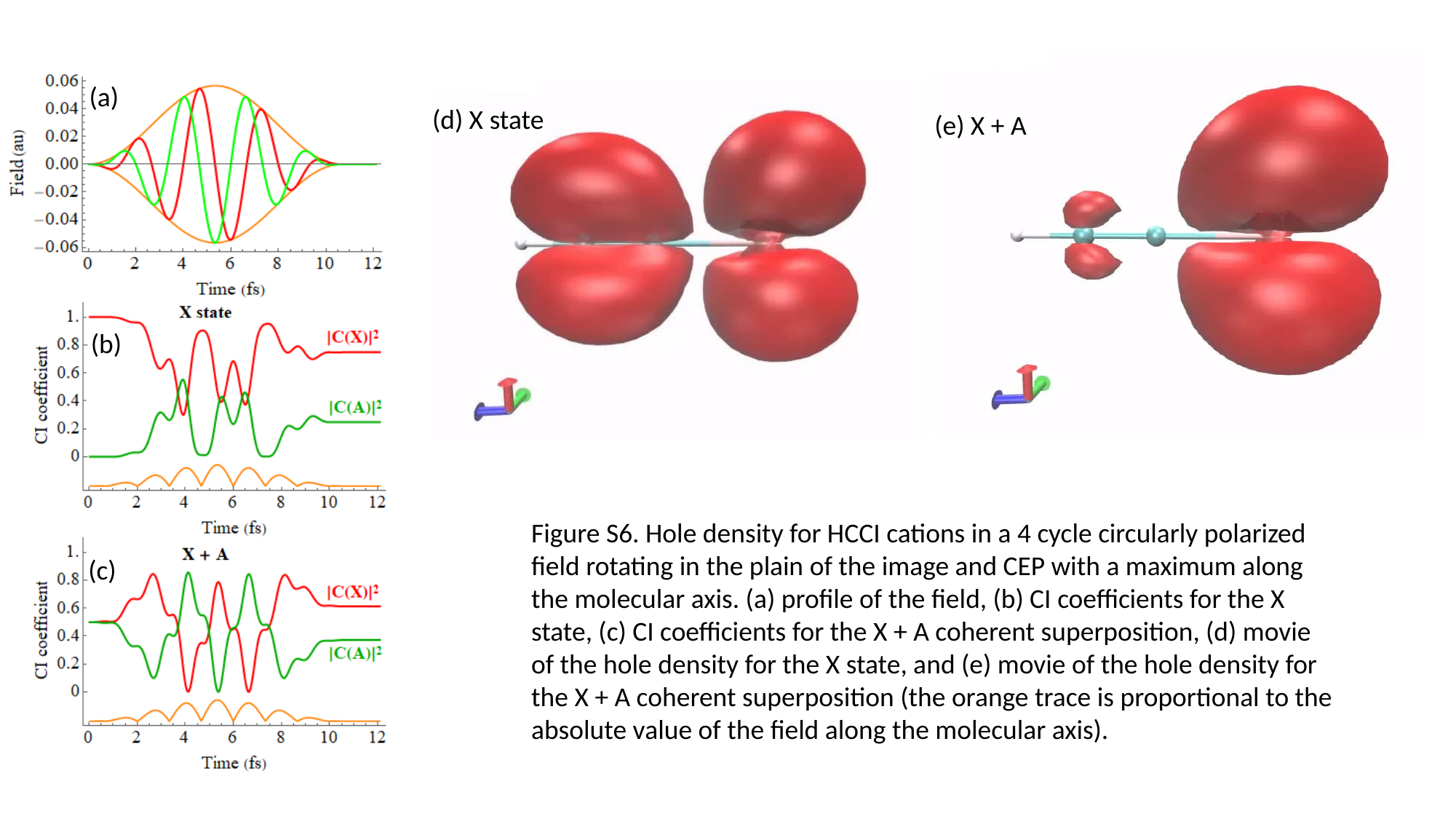

(e) X + A
(d) X state
(a)
(b)
(c)
Figure S6. Hole density for HCCI cations in a 4 cycle circularly polarized field rotating in the plain of the image and CEP with a maximum along the molecular axis. (a) profile of the field, (b) CI coefficients for the X state, (c) CI coefficients for the X + A coherent superposition, (d) movie of the hole density for the X state, and (e) movie of the hole density for the X + A coherent superposition (the orange trace is proportional to the absolute value of the field along the molecular axis).
